# Changes in frailty and depressive symptoms among middle-aged and older Chinese people: a nationwide cohort study

**DOI:** 10.1186/s12889-024-17824-3

**Published:** 2024-01-25

**Authors:** Ni Sang, Rong-chao Liu, Ming-hui Zhang, Zong-Xiao Lu, Zhen-Gang Wu, Meng-Yao Zhang, Bo-Han Li, Meng Wei, Hai-Feng Pan, Guo Cui Wu

**Affiliations:** 1 School of Nursing, Anhui Medical University, 15 Feicui Road, Hefei, Anhui 230032 China; 2 Inflammation and Immune Mediated Diseases Laboratory of Anhui Province, 81 Meishan Road, Hefei, Anhui China; 3 Department of Epidemiology and Biostatistics, School of Public Health, Anhui Medical University, Hefei, Anhui China

**Keywords:** Frailty, Depressive symptoms, Chinese middle-aged and older adults, CHARLS

## Abstract

**Background and aims:**

The older people bears a severe burden of disease due to frailty and depressive symptoms, however, the results of association between the two in the older Chinese people have been conflicting. Therefore, this study aimed to investigate the developmental trajectories and interactions of frailty and depressive symptoms in the Chinese middle-aged and older adults.

**Methods:**

The study used four waves of data from 2011, 2013, 2015 and 2018 in the China Health and Retirement Longitudinal Study (CHARLS) database, focused on middle-aged and older people ≥ 45 years of age, and analyzed using latent growth models and cross-lagged models.

**Results:**

The parallel latent growth model showed that the initial level of depressive symptoms had a significant positive predictive effect on the initial level of frailty. The rate of change in depressive symptoms significantly positively predicted the rate of change in frailty. The initial level of frailty had a significant positive predictive effect on the initial level of depressive symptoms, but a significant negative predictive effect on the rate of change in depressive symptoms. The rate of change in frailty had a significant positive predictive effect on the rate of change in depressive symptoms. The results of the cross-lagged analysis indicated a bidirectional causal association between frailty and depressive symptoms in the total sample population. Results for the total sample population grouped by age and gender were consistent with the total sample.

**Conclusions:**

This study recommends advancing the age of concern for frailty and depressive symptoms to middle-aged adults. Both men and women need early screening and intervention for frailty and depressive symptoms to promote healthy aging.

**Supplementary Information:**

The online version contains supplementary material available at 10.1186/s12889-024-17824-3.

## Introduction

China is the most populous country in the world and the country with the largest total older adults in the world [1]. According to the National Bureau of Statistics of China, by the end of 2022, the population aged 60 years and older in China was 280 million, accounting for 19.8% of the total population [2]. China is also aging significantly more rapidly than other countries, having entered a deeply aging society in only 21 years. The problems faced by the aging society are not completely the increased burden of old-age care, or face more severe health problems of the older people.

Older adults frequently experience frailty and depression, which come with a significant illness burden. Among them, frailty is a clinical state characterized by decreased physical physiological reserve, diminished stress resistance, increased vulnerability of the body and susceptibility to diseases [3]. Frailty is mostly related to advancing age, and its prevalence is fast increasing as the world’s aging population expands [4]. Frailty also increases the risk of adverse consequences of falls, hospitalization, and death in older adults [4]. Meanwhile, depression is regarded as the most common psychological problem in older adults, with up to one-third of all older adults experiencing depressive symptoms, yet its symptoms are often frequently simply ignored and inadequately treated [5], leading to family breakdown, disability, and the result of worsening disease, and increasing mortality [6].

According to previous studies, there is a high degree of overlap in diagnostic criteria for frailty and depression [7], such as fatigue, but they also have their own unique symptoms. And therefore, symptom overlap cannot accurately account for the association between these two diseases; there may be some connection between them. Several earlier studies have shown a heightened correlation between frailtyand depression [8, 9]. Frail older adults have an increased chance of experiencing depression, as do depressed patients [8]. In addition, results from a prospective cohort study pointed to frailty as an independent predictor of depressive symptoms in community-dwelling older adults [10]. Results from another prospective study supported the association of late-life depression with frailty in the following year [11]. Thus, there may be a causal relationship between frailty and depression. The strong association between frailty and depression can be explained in several ways: First both two share common risk factors, such as functional impairment and poor financial status [12–14]. Second, the two also share common pathophysiological pathways, such as the inflammatory system [15]. These studies suggested that frailty and depression may be “overlap syndromes” or that there may be some relationship between the two. In addition, because both frailty and early depressive symptoms were reversible, understanding the relationship between the two was important for early screening and treatment.

Considering the prevalence of frailty and depression in the older adults and the range of adverse consequences for older adults, it is critical to further explore the relationship between them. Two previous studies have been conducted in China on the relationship between frailty and depression in the Chinese older adults, but the findings on the association between the two were somewhat controversial. A bidirectional causal connection between frailty and depressive symptoms was demonstrated in a longitudinal study of older adults aged 70–84 in rural Jiangsu Province, China, using cross-lagged panel models [16]. The findings of this study may not be generally applicable to the whole older people in China because it was limited to a rural population of older adults aged 70 to 84 in one area. Another cohort study using the CHARLS database found that frailty was an independent predictor of subsequent depression among older (60 years and above) Chinese adults, but no association between frailty and depressive symptoms was found [17]. Additionally, the bidirectional causal association between frailty and depressive symptoms could not be investigated by the statistical method used in this study; it could only look at predictors. Consequently, in order to better understand how the two interact dynamically and how this affects the process of personal growth, more potent statistical methods should be used.

Frailty is an age-related geriatric syndrome [18], and a recent study reported that the prevalence of pre-frailty and frailty in people aged 40–49 years was 45% and 1.4%, respectively, equivalent to those aged 70–75 years [19] suggesting that we should start preventing frailty at the age of 40. Additionally, a recent study has revealed that middle-aged persons in the UK population have a more significant link between frailty and mental health than older adults [20]. In addition, Li et al [21] recruited 1789 community-dwelling older adults from eastern China. Using the frailty phenotype and the 5-item Geriatric Depression Scale to assess frailty and depressive symptoms, respectively, the results also showed that frailty was more likely to cause depressive symptoms in young adults than in older adults. This showed that the association between frailty and depressive symptoms may be more pronounced in middle-aged people. This may be because enhanced emotion regulation capacity with age may contribute to buffering the adverse effects of frailty on emotional well-being, and therefore the relationship between frailty and depressive symptoms becomes weaker with age. However, there are currently no study on the association between frailty and depressive symptoms in the Chinese middle-aged population, and there is a dearth of knowledge on the intervention variables that target frailty in middle-aged adults.

Currently, gender differences in the relationship between frailty and depressive symptoms are controversial. Liu et al. ‘s cohort study of 7609 community-dwelling older adults found no gender-specific association between depressive symptoms and frailty [22]. However, the results of Wen et al. ‘s meta-analysis of frailty and depressive symptoms in community-dwelling older adults showed that older men with depression were more likely to be frail than older women with depression [23]. This is consistent with the results of a previous study of older Chinese adults, which also concluded that there are gender differences in the relationship between physical frailty and depressive symptoms [24]. There are few studies on the role of gender in the relationship between depression and frailty. Therefore, it is necessary to investigate gender differences in the relationship between frailty and changes in depressive symptoms.

Therefore, the purpose of this study was to further examine: (1) the trajectory of frailtyand depressive symptoms in the Chinese middle-aged and older adults. (2) The causal direction of frailty and depressive symptoms in the Chinese middle-aged and older adults. (3) To investigate whether there are gender and age differences in the relationship between frailty and depressive symptoms in the middle-aged and older Chinese population. This information can serve as a scientific guide for the early use of preventative strategies and therapeutic regimens for incapacitating and depressed symptoms in the Chinese middle-aged and older adults.

Therefore, the main marginal contributions of this study are (1) The current study makes up for this gap by investigating the relationship between frailty and depressive symptoms. (2) In the past, most of the studies on the relationship between frailty and depressive symptoms in China started from the perspective of the older adults, this study added the middle-aged population and analyzed them by gender, making the content of the relationship between frailty and depressive symptoms richer and clearer.

## Methods

### Data source and participants

The China Health and Retirement Longitudinal Study (CHARLS) was the source of all the information used in this study [25]. CHARLS is a high-quality public database designed to investigate families and individuals of people aged 45 years and older, using multistage PPS sampling, a household survey of middle-aged and older adults in 28 provincial administrative regions of China covering 450 communities (villages) in 150 counties and districts of 28 provinces across the country, including personal basic information, family structure and economic support, health status and physical measurements [25]. The baseline survey was conducted in 2011 and tracked biennially, with participants followed up in 2013, 2015, and 2018, and phase 4 data have now been published and generally represent the Chinese older adults. CHARLS was approved by the Biomedical Ethics Committee of Peking University and informed consent was obtained from all participants.

Data from 2011 (first survey, 17,708 participants), 2013 (second survey), 2015 (third survey) and 2018 ( fourth survey) CHARLS database were used in this study. The following were the inclusion requirements for participants in this study: (1) Age ≥ 45 years; (2) Complete general demographic data, including age, gender, smoking, drinking, education, and marital status; (3) Complete information on frailty and depressive symptoms could be provided; (4) Participants participated in all four surveys.

**Fig. 1:**
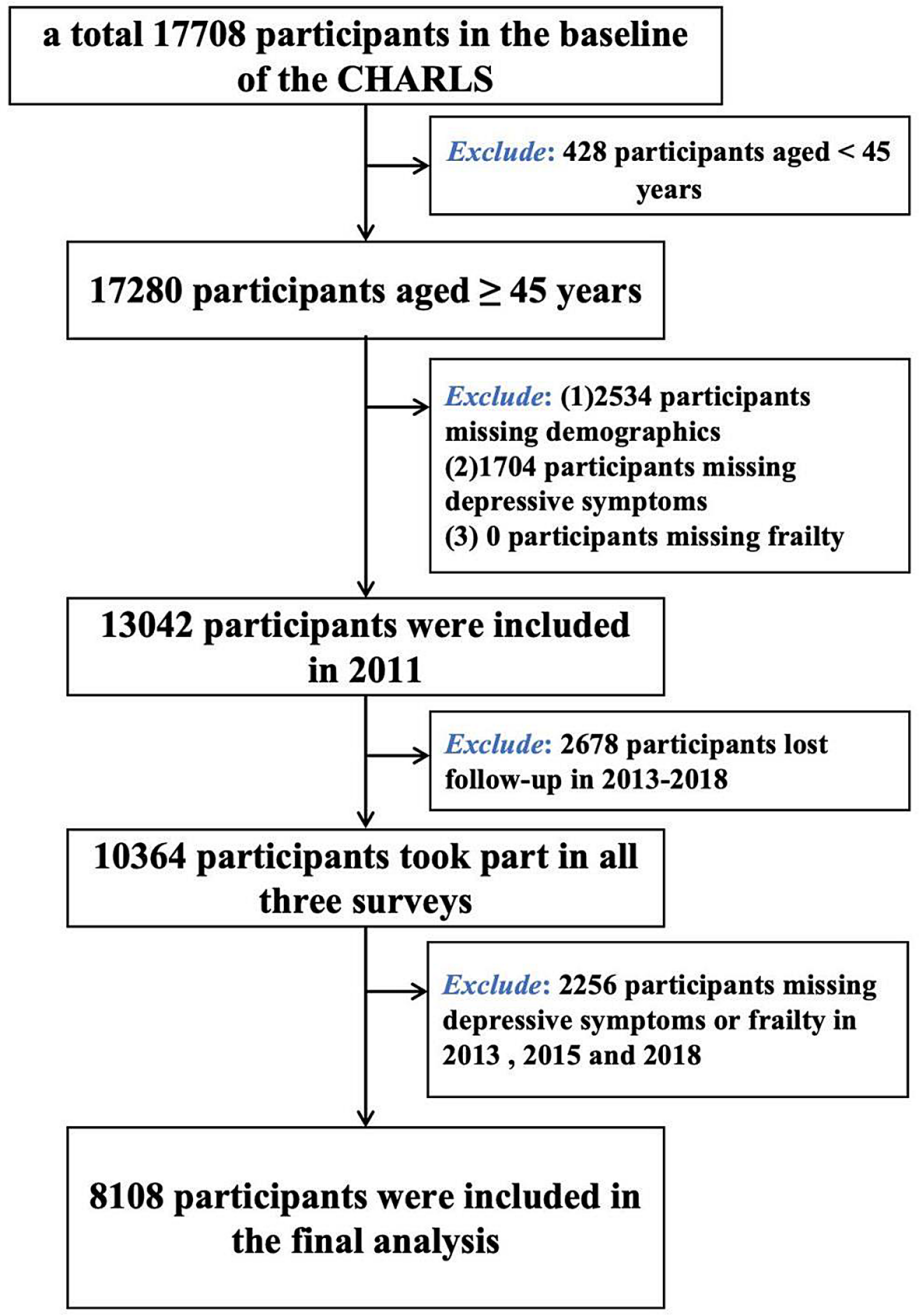
Flowchart of participant selection

Figure 1 shows the selection process for study participants. A total of 17,708 participants were available at baseline (2011), 17,280 participants were ≥ 45 years of age, a final total of 13,042 participants were finally included in the trial at baseline after excluding 4238 people who had missing demographic, depression symptoms, and frailty data at baseline, 10,364 participants attended all three surveys after excluding 2678 participants who were lost during follow-up in 2013, 2015 and 2018, 2256 participants with missing depressive symptoms or frailty data, and the study eventually comprised 8108 participants altogether **(**Fig. 1**)**.

### Measurements

#### Frailty

The frailty index (FI) was measured to estimate the frailty, which calculated the accumulation of age-related health defects, and its index can be programmed and freely constructed according to needs on the basis of following the health defect selection principle, but it should contain at least 30 health defect items [26]. In this study, 40 indexes were selected to construct the FI assessment scale according to the criteria for constructing FI [27]: 6 items in Basic Activity of Daily Living (BADL), 5 entries for Instrumental Activities of Daily Living Scale (IADL), hearing disorder, visual impairment, language disorder, 14 chronic diseases (Hypertension, Dyslipidemia, Diabetes or high blood sugar, Cancer or malignant tumor, Chronic lung diseases, Liver disease, Heart attack, Stroke, Kidney disease, Stomach or other digestive diseases, Emotional, nervous, or psychiatric problems, Memory-related disease, Arthritis or rheumatism, Asthma), 2 disabilities (Physical disabilities and Brain damage/mental retardation), Self-assessed health status and health changes, 3 mobility, 5 muscle performance and cognitive function measured using telephone interview for cognitive status-modified (TICS-m). TICS-m includes daily memory ability, immediate memory ability, delayed memory ability, calculation and drawing ability, with a total of 21 questions, and a total score of 0 to 21 points, with higher scores having a worser cognitive function, and health deficit scores taking the value of the actual score/21 (the list of frailty indexes was shown in Supplementary Table 1). The evaluation of the above dimensions assigned health variables 0, 1 (0 = no health deficit, 1 = health deficit) according to the type of variable, etc. FI was calculated by dividing the health deficit score by the total number of participants (40 in this study), which ranged from 0 to 1, with larger values indicating more weakness. FI has already been used in several CHARLS studies [28, 29].

#### Depressive symptoms

Depressive symptoms in CHARLS were assessed using the 10-item Center for Epidemiological Studies Depression Scale (CESD-10), which used a Likert score of 0 to 3 to assess the frequency of symptoms experienced by participants over the past 1 week, with the following response options: Rarely or none of the time represented ≤ 1 day and was assigned a value of 0; Some or a little of the time represented 1–2 days and was assigned a score of 1; Occasionally or a moderate amount of the time represents 3–4 days and was assigned a value of 2; Most or all of the time represented 5–7 days and was assigned a value of 3. Among them, questions 5 and 8 were entitled reverse scoring questions, and responses were assigned reverse. The total score on the scale ranged from 0 to 30, with higher scores indicating more severe depressive symptoms. CES-D-10 had been validated in the older Chinese population [30]. In this study, the CESD Cronbach’s α in 2011 was 0.805, KMO = 0.880, Bartlett ‘s spherical test P < 0.001, the CESD Cronbach’ s α in 2013 was 0.805, KMO = 0.880, Bartlett ‘s spherical test P < 0.001, the CESD Cronbach’s α in 2015 was 0.798, KMO = 0.883, Bartlett ‘s spherical test P < 0.001, the CESD Cronbach’ s α in 2018 was 0.752, KMO = 0.893, Bartlett ‘s spherical test P < 0.001, and the reliability and validity of the CES-D-10 scale measured four times were all Excellent.

#### Demographic variables

Demographic variables in this study included age; sex (male; female); Marital status (Married with spouse present; Married but not living with spouse temporarily for reasons such as work; Separated; Divorced; Widowed; Never married); Education (No formal education (illiterate); Elementary school or below; Middle school and above); Smoking was assessed based on whether or not smoking had occurred (yes; no); Alcohol consumption was assessed according to whether or not alcohol was consumed in the past year and frequency of alcohol consumption (Drink more than once a month; Drink but less than once a month; None of these).

### Statistical analysis

Firstly, Means ± Standard Deviations (M ± SD) and frequencies (percentages) were used to describe the basic characteristics of study participants. Then, the correlation between frailty and depressive symptoms was determined using Spearman correlation analysis (frailty and depressive symptoms were tested to be skewed distributions). Then Latent Growth Modeling (LGM) was constructed to explore the trends of frailty and depression symptoms in the middle-aged and older adults. And the total sample population was divided according to gender to construct latent growth models, respectively. LGM is an important statistical method for exploring the developmental trajectories of variables and is generally modeled by data from three or more time points [31]. In LGM, the intercept represents the initial level of the variable and the slope represents the rate of change of the variable [32]. Finally, a cross-lagged panel model (CLPM) was created to see if there was a causal relationship between frailty and depressive symptoms in the older Chinese population. And we divided the total sample into two groups: male and female and established CLPM separately. CLPM was a longitudinal analytical model that investigated the inter-predictive relationship or quasi-causal relationship between variables [33]. According to the measurement of two variables at different time points, the estimated path coefficients of the influence of the pretest variables on the posttest variables had a temporal order relationship, which was in line with the principle of epidemiological causal inference. Several correlation coefficients were obtained by calculation. Among them, comparing the absolute value of standardized coefficients could investigate the strength of the causal relationship between variables. Model goodness of fit was assessed using the following indices [34]: Comparative fit index (CFI), Tucker-Lewis index (TLI), Root mean square error of approximation (RMSEA) and Standardized root mean square residual error (SRMR) were compared for evaluation. Where CFI > 0.9, TLI > 0.9, RMSEA < 0.08, SRMR < 0.08 indicate a good model fit. Considering multiple repeated measurements, *P* < 0.01 was considered statistically significant. R 4.2.1 was used to accomplish the data cleaning. R software was utilized for the remainder of the statistical study, with the exception of Mplus, which was employed to create the latent growth model. The R packages used for these analyses were lavaan, tidySEM, and corrplot.

## Results

### Descriptive analysis

A total of 8108 participants were included in the study in the total sample, including 4996 middle-aged adults aged 45–59 years and 3112 older adults ≥ 60 years. 48.0% of the participants in the total sample were male, 3186 participants smoked, and 65.70% never drank alcohol. By education level, only 22.50% of participants in the total sample were illiterate. Table 1 showed the basic information for the total sample, the 45–59 age group (middle-aged) and the ≥ 60 age group (older) (Table 1**)**.

**Table 1:** Descriptive statistics for 8108 study participants

Variables		Total	45–59	≥ 60
N		8108	4996	3112
Age (M ± SD)		57.48 ± 8.122	52.23 ± 4.411	65.89 ± 5.064
Gender	Male	3892 (48.0%)	2269 (45.4%)	1623 (52.2%)
	Female	4216 (52.0%)	2727 (54.6%)	1489 (47.8%)
Smoking	Yes	3186 (39.3%)	1836 (36.7%)	1350 (43.4%)
	No	4922 (60.7%)	3160 (63.3%)	1762 (56.6%)
Drinking	Drink more than once a month	2123 (26.2%)	1308 (26.2%)	815 (26.2%)
	Drink but less than once a month	657 (8.1%)	435 (8.7%)	222 (7.1%)
	None of these	5328 (65.7%)	3253 (65.1%)	2075 (66.7%)
Education	No formal education (illiterate)	1822 (22.5%)	960 (19.2%)	862 (27.7%)
	Elementary school or below	3398 (41.9%)	1829 (36.6%)	1569 (50.4%)
	Middle school school and above	2888 (35.6%)	2207 (44.2%)	681 (21.9%)
Marital status	Married with spouse present	7031 (86.7%)	4483 (89.7%)	2548 (81.9%)
	Married but not living with spouse temporarily for reasons such as work	336 (4.1)	261 (5.2%)	75 (2.4%)
	Separated	26 (0.3%)	10 (0.2%)	16 (0.5%)
	Divorced	52 (0.6%)	32 (0.6%)	20 (0.6%)
	Widowed	612 (7.5%)	179 (3.6%)	433 (14.9%)
	Never married	51 (0.6%)	31 (0.6%)	20 (0.6%)
FIT1		0.091 ± 0.070	0.081 ± 0.064	0.107 ± 0.075
FIT2		0.052 ± 0.055	0.046 ± 0.050	0.063 ± 0.061
FIT3		0.072 ± 0.063	0.064 ± 0.056	0.086 ± 0.070
FIT4		0.096 ± 0.082	0.083 ± 0.072	0.118 ± 0.091
DST1		8.13 ± 6.226	7.80 ± 6.117	8.68 ± 6.361
DST2		7.77 ± 5.750	7.68 ± 5.741	7.93 ± 5.762
DST3		8.01 ± 6.408	7.82 ± 6.330	8.32 ± 6.519
DST4		9.61 ± 8.061	9.19 ± 7.507	10.28 ± 8.837

*Note* M = mean; SD = Standard Deviation; FIT1 = frailty index in 2011; FIT2 = frailty index in 2013; FIT3 = frailty index in 2015; FIT4 = frailty index in 2018; DST1 = depressive symptoms in 2011; DST2 = depressive symptoms in 2013; DST3 = depressive symptoms in 2015; DST4 = depressive symptoms in 2018

### Correlation analysis between frailty and depressive symptoms

**Fig. 2:**
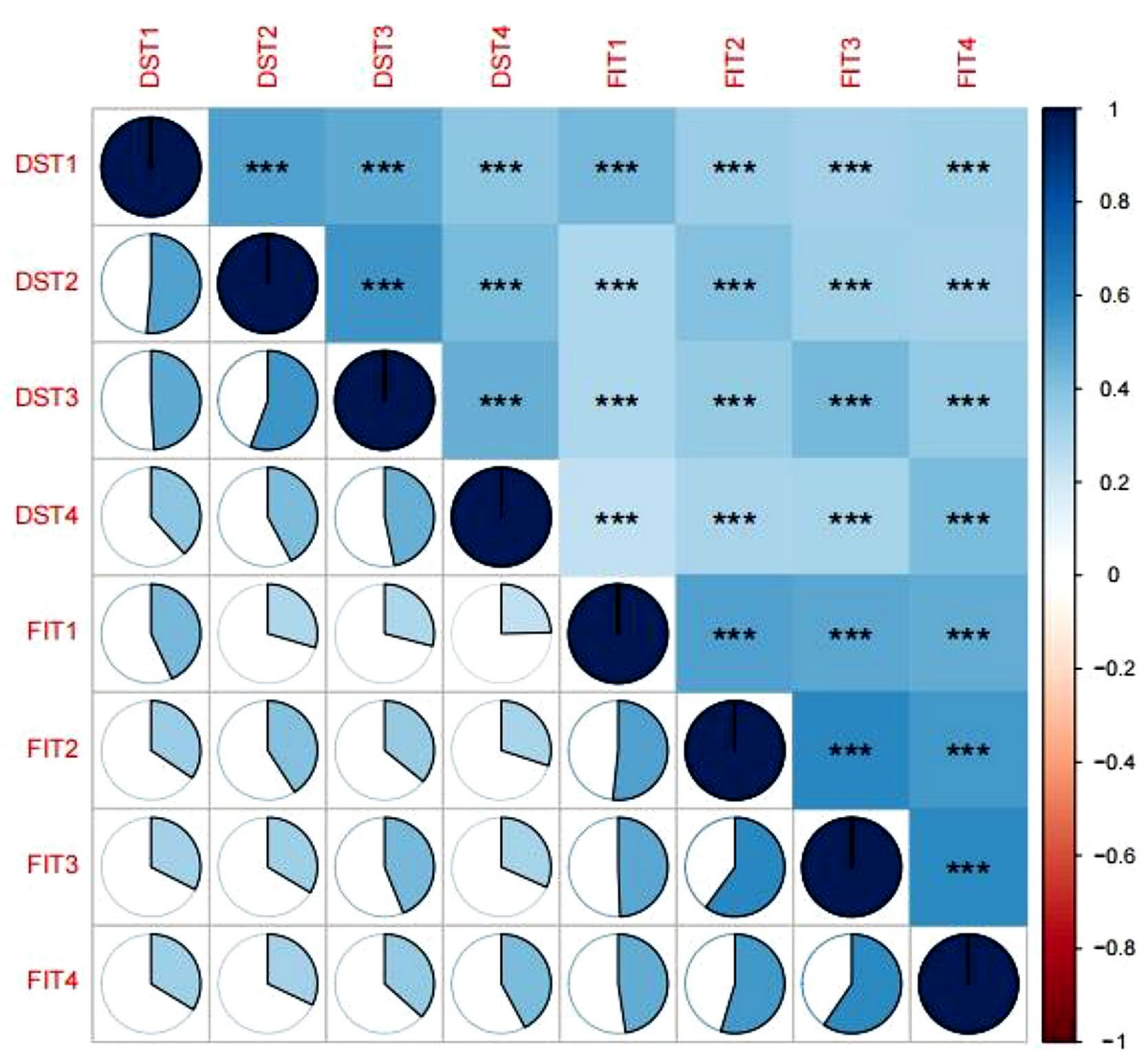
Spearman correlation between frailty and depressive symptoms at T1, T2, T3 and T4 in the total sample population. *Note* FIT1 = frailty index in 2011; FIT2 = frailty index in 2013; FIT3 = frailty index in 2015; FIT4 = frailty index in 2018; DST1 = depressive symptoms in 2011; DST2 = depressive symptoms in 2013; DST3 = depressive symptoms in 2015; DST4 = depressive symptoms in 2018; ***:*P* < 0.001

The results of the correlation analysis were shown in Fig. 2. There was a significant positive correlation between frailty and depressive symptoms at each time point in the total sample (all *P* < 0.001), and a significant positive correlation between frailty and depressive symptoms at each time in both middle-aged and older groups at each time (all *P* < 0.001) (Supplementary Figs. 1&2). There was also a significant positive correlation between frailty and depressive symptoms at each time in both male and female groups (all *P* < 0.001) (Supplementary Figs. 3&4).

### Developmental trajectory of frailty and depressive symptoms

An unconditional linear growth model for the frailty of the total sample population was first constructed to test the developmental trajectory of the frailty. Its model fitting results were not very satisfactory. The starting degree of fading was unrelated to its rate of change during the four measurements, which revealed a significant increasing trend in fading (*β* = 0.006, *P* < 0.001). Unconditional linear growth models for frailty in the middle-aged and older adultss were constructed separately by subgroups, and the results were consistent with those of the total sample (Table 2). Unconditional linear growth models of frailty were constructed separately for male and female subgroups, and the results were consistent with those for the total sample (Table 2).

**Table 2:** Unconditional linear growth model of frailty and depressive symptoms

	Model Fit Indices						Intercept			Slope			Slope with intercept		
	*χ* ^*2*^	*df*	CFI	TLI	RMSEA	SRMR	*β*	*se*	*P*	*β*	*se*	*P*	*β*	*se*	*P*
Total															
Frailty	2175.117	5	0.595	0.514	0.231	0.163	0.061	0.001	< 0.001	0.006	0.000	< 0.001	0.000	0.000	< 0.001
Depressive symptoms	271.914	5	0.949	0.939	0.081	0.042	7.684	0.061	< 0.001	0.355	0.022	< 0.001	0.259	0.199	0.193
45–59 year															
Frailty	1238.038	5	0.580	0.506	0.222	0.157	0.055	0.001	< 0.001	0.005	0.001	< 0.001	0.000	0.000	< 0.001
Depressive symptoms	102.607	5	0.969	0.963	0.063	0.036	7.471	0.077	< 0.001	0.357	0.028	< 0.001	0.304	0.247	0.218
≥ 60 year															
Frailty	983.319	5	0.552	0.463	0.251	0.183	0.072	0.001	< 0.001	0.008	0.001	< 0.001	0.001	0.000	< 0.001
Depressive symptoms	204.275	5	0.906	0.887	0.113	0.053	8.038	0.103	< 0.001	0.336	0.038	< 0.001	0.076	0.333	0.820
Male															
Frailty	919.287	5	0.546	0.455	0.217	0.174	0.053	0.001	< 0.001	0.005	0.000	< 0.001	0.000	0.000	< 0.001
Depressive symptoms	106.588	5	0.952	0.943	0.072	0.039	6.719	0.079	< 0.001	0.257	0.030	< 0.001	0.429	0.245	0.080
Female															
Frailty	1091.031	5	0.613	0.536	0.242	0.160	0.066	0.001	< 0.001	0.007	0.000	< 0.001	0.000	0.000	< 0.001
Depressive symptoms	75.836	5	0.979	0.975	0.062	0.035	8.550	0.096	< 0.001	0.300	0.032	< 0.001	-0.331	0.277	0.232

*Note* χ^2^: Chi-Square; se = standard error

Following that, a well-fitting unconditional linear growth model for depressive symptoms in the entire sample group was developed. The initial level of the intercept was 7.684. Levels of depressive symptoms were stable across the four measurements (*β* = 0.355, *P* = 0.529). The intercept did not correlate with the slope (*β* = 0.259, *P* = 0.193), which suggested that individual initial depressive symptom levels do not correlate with their depression level growth rate. (Table 2). Unconditional linear growth models of depressive symptoms were constructed separately for the middle-aged, older people, male, and female groups, and the results were consistent with those for the total sample. Therefore, the unconditional linear growth model of frailty and depressive symptoms for the total sample was used as a discussion model in this study.

### A parallel latent growth model for frailty and depressive symptoms

**Fig. 3:**
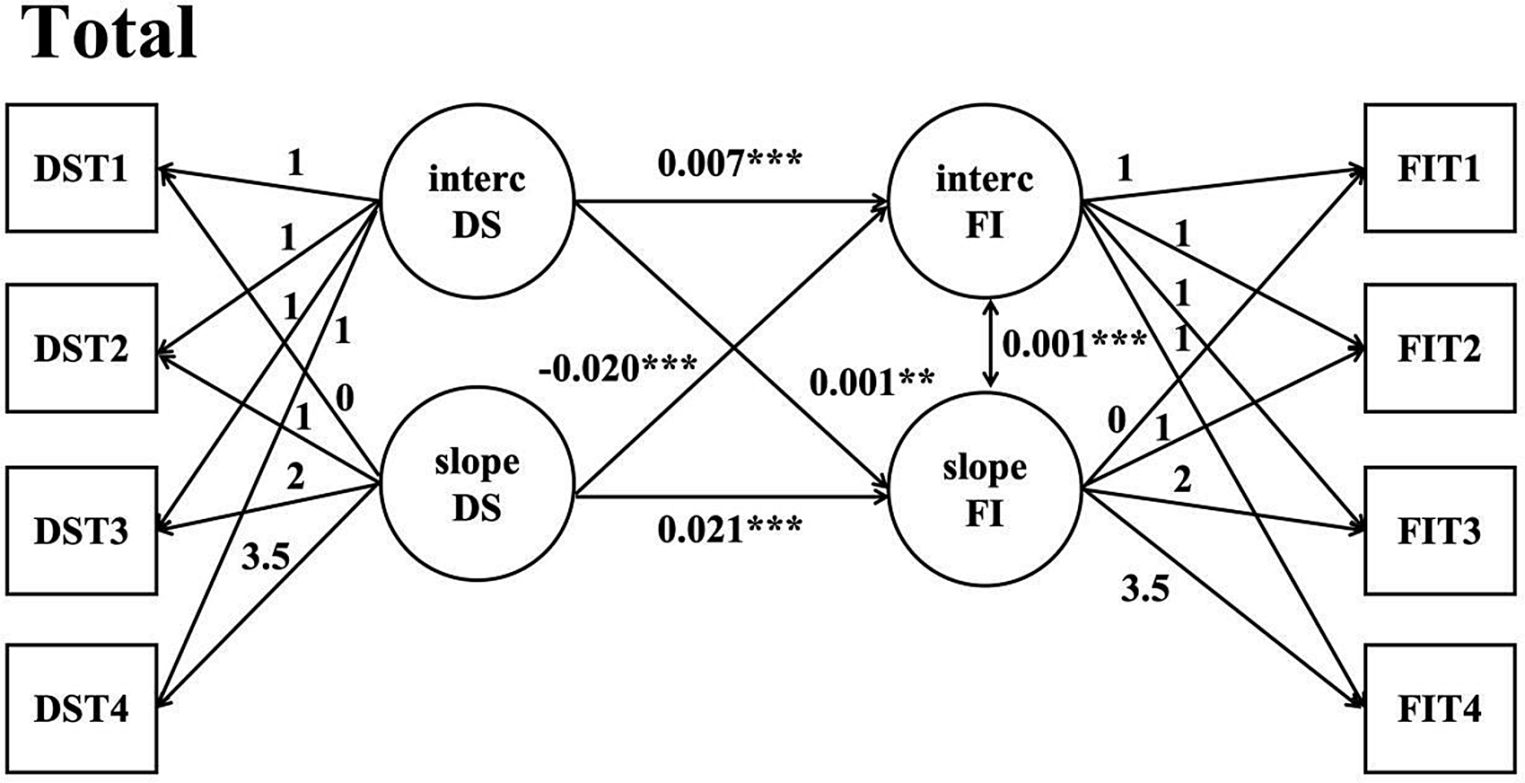
A parallel latent growth model for depressive symptoms on frailty in the total sample population. *Note* DS = depressive symptoms; FI = frailty index; FIT1 = frailty index in 2011; FIT2 = frailty index in 2013; FIT3 = frailty index in 2015; FIT4 = frailty index in 2018; DST1 = depressive symptoms in 2011; DST2 = depressive symptoms in 2013; DST3 = depressive symptoms in 2015; DST4 = depressive symptoms in 2018; Adjusting covariates: Age; Education; Marital status; Smoking; Drinking; Gender; ***: *P* < 0.001; **: *P* < 0.01

In order to investigate the relationship between the initial level of depressive symptoms, the development trajectory of depressive symptoms, the initial level of frailty and the development trajectory of frailty. First we constructed a parallel latent variable growth model of depressive symptoms on the direction of frailty and adjusted for covariates, whose parameters in the model were in the supplementary materials (supplementary Table 2). Model fitting indexes: *χ*^*2*^ = 4334.951, *df* = 47, *P* < 0.05, CFI = 0.792, TLI = 0.664, AIC = 109776.31, BIC = 110091.359, RMSEA = 0.106. The regression coefficients from the intercept factor of depressive symptoms to the intercept factor and slope factor of frailty were 0.007 (*P* < 0.001) and 0.001 (*P* < 0.001), respectively, indicating that the initial level of depressive symptoms had a significant positive predictive effect on the initial level of frailty and a significant positive predictive effect on the rate of change of frailty. The regression coefficients of the slope factor of depressive symptoms on the intercept factor and slope factor of frailty were − 0.020 (*P* < 0.001) and 0.021 (*P* < 0.001), and the results showed that the rate of change of depressive symptoms had a significant negative predictive effect on the initial level of frailty, but a significant positive predictive effect on the rate of change of frailty. The regression coefficient of the slope factor for the frailty intercept factor was 0.001 (*P* < 0.001), indicating that there was a positive association between the initial level of frailty and the rate of development (Fig. 3)

**Fig. 4:**
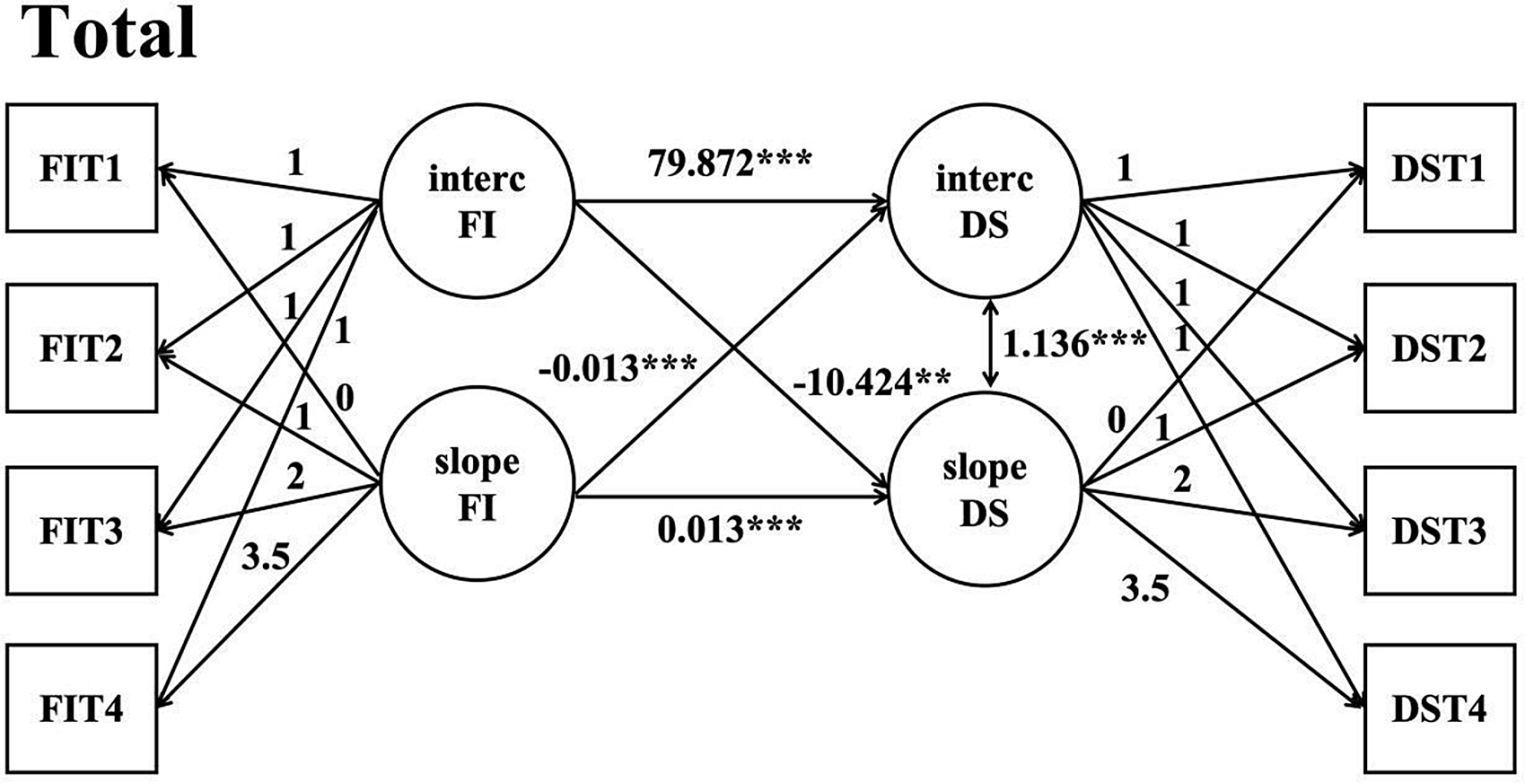
A parallel latent growth model for frailty on depressive symptoms in the total *Note* DS = depressive symptoms; FI = frailty index; FIT1 = frailty index in 2011; FIT2 = frailty index in 2013; FIT3 = frailty index in 2015; FIT4 = frailty index in 2018; DST1 = depressive symptoms in 2011; DST2 = depressive symptoms in 2013; DST3 = depressive symptoms in 2015; DST4 = depressive symptoms in 2018; Adjusting covariates: Age; Education; Marital status; Smoking; Drinking; Gender; ***: *P* < 0.001; **:*P* < 0.01

We then constructed a parallel latent growth model for the direction of depression symptoms by frailty and adjusted for covariates, and the parameters of covariates in the model were in the Supplementary materials (Supplementary Table 2). Model fitting indexes*χ*^*2*^ = 4508.457, *df* = 47, *P* < 0.05, CFI = 0.784, TLI = 0.650, AIC = 110129.197, BIC = 110444.224, RMSEA = 0.108. The results suggested that the model fitting was acceptable. The regression coefficients of the intercept factor of frailty to the intercept factor and slope factor of depressive symptoms were 79.872 (*P* < 0.001) and − 10.424 (*P* < 0.001), respectively, indicating that the initial level of depressive symptoms had a significant positive predictive effect on the initial level of frailty and a significant negative predictive effect on the rate of change of frailty. The regression coefficients of the intercept factor and slope factor of the frailty slope factor on depressive symptoms were − 0.013 (*P* < 0.001) and 0.013 (*P* < 0.001), and the results showed that the rate of change of frailty had a significant negative predictive effect on the initial level of depressive symptoms, but a significant positive predictive effect on the rate of change of depressive symptoms. The regression coefficient of the intercept factor on the slope factor for depressive symptoms was 1.136 (*P* < 0.001), indicating that there was a positive association between the initial level of depressive symptoms and the rate of development (Fig. 4).

Then we constructed parallel latent growth models for the middle-aged and older adultss separately. A model for the middle-aged population is presented in the supplementary materials. The fitting indexes of parallel latent growth model of depressive symptoms on the direction of frailty were *χ*^*2*^ = 2341.24, *df* = 43, *P* < 0.05, CFI = 0.804, TLI = 0.690, AIC = 62956.301, BIC = 63223.473, RMSEA = 0.103, SRMR = 0.068. The results suggested that the fitting results of the model were acceptable. The model showed mostly consistent results with those for the total sample. (Supplementary Fig. 5). Except for the model where the regression coefficients of the intercept factors for depressive symptoms and frailty on the slope factor for frailty were both 0, this suggested that the initial levels of depressive symptoms and frailty are not associated with the rate of frailty development in middle-aged populations. The fitting indexes of parallel latent growth model of frailty on the direction of depressive symptoms were *χ*^*2*^ = 2420.944, *df* = 43, *P* < 0.05, CFI = 0.797, TLI = 0.679, AIC = 63122.138, BIC = 63389.310, RMSEA = 0.105, SRMR = 0.070. The results suggested that the model fitting results were acceptable. The model showed results consistent with those for the total sample (Supplementary Fig. 6). Models for the older adults are presented in the supplementary materials, The fitting indexes of parallel latent variable growth model of depressive symptoms on the direction of frailty were *χ*^*2*^ = 1958.483, *df* = 43, *P* < 0.05, CFI = 0.756, TLI = 0.614, AIC = 46124.015, BIC = 46371.779, RMSEA = 0.120, SRMR = 0.080. The results suggested that the fitting results of the model were acceptable. Except for the intercept factor for depressive symptoms in this model, the regression coefficient for the slope factor for frailty was 0. 001 (*P* > 0.05), which suggested that the initial level of depressive symptoms is not associated with the rate of decline development in the older people (Supplementary Fig. 7). Fitting indexes of parallel latent growth model of frailty on depressive symptom direction: *χ*^*2*^ = 2060.614, *df* = 43, *P* < 0.05, CFI = 0.743, TLI = 0.594, AIC = 46311.037, BIC = 46558.800, RMSEA = 0.123, SRMR = 0.083, and the results suggested that the model fitting results were acceptable. The model showed results consistent with those for the total sample (Supplementary Fig. 8).

In addition, this study also divided the total sample population according to gender to construct parallel latent growth models, respectively. Models for the male group were presented in the supplementary materials. The fitting indexes of parallel latent growth model of depressive symptoms on the direction of frailty were *χ*^*2*^ = 1984.986, *df* = 43, *P* < 0.05, CFI = 0.762, TLI = 0.624, AIC = 49193.565, BIC = 49450.499, RMSEA = 0.108, SRMR = 0.076 (Supplementary Fig. 9). The results suggested that the fitting results of the model were acceptable. The model showed mostly consistent results with those for the total sample. Except for the intercept factor for depressive symptoms, which had a regression coefficient of 0 for the slope factor for frailty, the initial level of depressive symptoms was therefore independent of frailty on the rate of development in the male group. Fitting indexes of parallel latent growth model of asthenia on depressive symptom direction: *χ*^*2*^ = 2077.250, *df* = 43, *P* < 0.05, CFI = 0.751, TLI = 0.606, AIC = 49383.942, BIC = 49640.876, RMSEA = 0.110, SRMR = 0.079 (Supplementary Fig. 10). The results suggested that the model fitting results were acceptable. The model showed results consistent with those for the total sample. The model of female group is shown in the supplementary materials, and the parallel latent growth model fitting index of depressive symptoms on the direction of frailty: *χ*^*2*^ = 1911.751, *df* = 43, *P* < 0.05, CFI = 0.816, TLI = 0.710, AIC = 49743.520, BIC = 49998.546, RMSEA = 0.108, SRMR = 0.070, and the results suggested that the model fitting results were acceptable Supplementary Fig. 11). The model showed mostly consistent results with those for the total sample. Except that the regression coefficient of the intercept factor of depressive symptoms on the slope factor of frailty in this model was 0, this indicated that in the female group, the initial level of depressive symptoms was not associated with the rate of frailty development, which was consistent with the results in the male group. Fitting indexes of parallel latent variable growth model of weakness on depressive symptom direction: *χ*^*2*^ = 2060.614, *df* = 43, *P* < 0.05, CFI = 0.743, TLI = 0.594, AIC = 46311.037, BIC = 46558.800, RMSEA = 0.123, SRMR = 0.083, and the results suggested that the model fitting results were acceptable Supplementary Fig. 12). The model showed results consistent with those for the total sample.

### Cross-lagged panel analysis

**Fig. 5:**
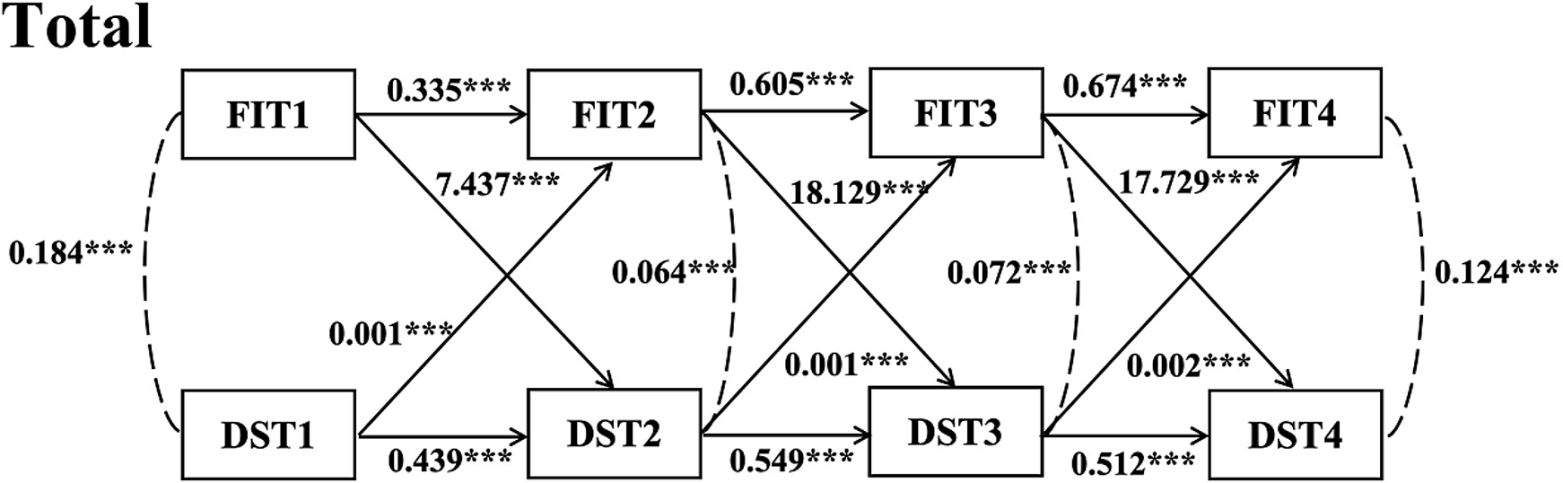
Cross-lagged Model for Frailty and Depressive Symptoms for the Total Sample. *Note* FIT1 = frailty index in 2011; FIT2 = frailty index in 2013; FIT3 = frailty index in 2015; FIT4 = frailty index in 2018; DST1 = depressive symptoms in 2011; DST2 = depressive symptoms in 2013; DST3 = depressive symptoms in 2015; DST4 = depressive symptoms in 2018; Adjusting covariates: Age; Education; Marital status; Smoking; Drinking; Gender; ***:*P* < 0.001

Figure 5 showed a cross-lagged model built at three-time points: frailty and depressive symptoms, which fits well (CFI = 0.900, TLI = 0.767, RMSEA = 0.157, SRMR = 0.083). Cross-lagged path analysis showed significant correlations between frailty and depressive symptoms at T1, T2, and T3. Depressive symptoms (*β* = 7.437, *P* < 0.001) and frailty (*β* = 0.335, *P* < 0.001) at moment T2 were found to significantly predict by frailty at moment T1. Depressive symptoms at T1 significantly predicted frailty (*β* = 0.001, *P* < 0.001) and depressive symptoms (*β* = 0.439, *P* < 0.001) at T2. Frailty at T2 significantly predicted depressive symptoms (*β* = 18.129, *P* < 0.001) and frailty (*β* = 0.605, *P* < 0.001) at T3. Depressive symptoms at T2 significantly predicted frailty (*β* = 0.001, *P* < 0.001) and depressive symptoms (*β* = 0.549, *P* < 0.001) at T3. Frailty at T3 significantly predicted depressive symptoms (*β* = 17.729, *P* < 0.001) and frailty (*β* = 0.674, *P* < 0.001) at T4. Depressive symptoms at T3 significantly predicted frailty (*β* = 0.001, *P* < 0.001) and depressive symptoms (*β* = 0.549, *P* < 0.001) at T4.

Cross-lagged models were constructed to investigate the inter-predictive relationship between frailty and depressive symptoms by grouping the total sample by age. The model fit was good for the middle-aged group (45–59 years) (CFI = 0.898, TLI = 0.763, RMSEA = 0.157, SRMR = 0.084). Model fit was acceptable for the older group (≥ 60 years) (CFI = 0.906, TLI = 0.781, RMSEA = 0.151, SRMR = 0.080). The outcomes of the cross-lagged model in the categories of middle-aged and older people were in line with the outcomes of the cross lagged model in the entire sample (Supplementary Figs. 13&14).

Then, we divided the total sample by gender and constructed cross-lagged models to investigate the inter-predictive relationship between frailty and depressive symptoms. The model fit was good for the female group (CFI = 0.900, TLI = 0.767, RMSEA = 0.157, SRMR = 0.082). Model fit was acceptable for the male group (CFI = 0.894, TLI = 0.752, RMSEA = 0.155, SRMR = 0.084). Cross-lagged model results for male and female groups were consistent with cross-lagged model results for the total sample (Supplementary Figs. 15&16).

Because the cross-lagged model results for the middle-aged group, the older group, the male group and the female group were consistent with the cross-lagged model results for the total sample. Therefore, the cross-lagged model for the total sample was taken as the model investigated in this study.

## Discussion

In this study, we investigated the changes of frailty and depressive symptoms in the middle-aged and older Chinese population, as well as the interaction between frailty and depressive symptoms. The main findings suggested that, first, both frailty and depressive symptoms increased during the four measurements. Second, the higher the initial level of depressive symptoms, the higher the initial level of frailty. The faster the rate of change of depressive symptoms, the faster the rate of change of frailty. Third, the higher the initial level of frailty, the higher the initial level of depressive symptoms, the slower the rate of change. The faster the rate of change of frailty, the lower the initial level of depressive symptoms, the faster the rate of change. Fourth, there was a bidirectional causal relationship between frailty and depressive symptoms. Finally, the bidirectional causality between frailty and depressive symptoms was independent of age and gender.

The faster the rate of change in depressive symptoms, the faster the rate of change in frailty. The reason for this may be that depressive symptoms were positively correlated with frailty, and the level of frailty increases as depressive symptoms increase. The higher the initial level of frailty, the slower the rate of change in depressive symptoms, which may be because the higher the initial level of frailty, the higher the initial level of depressive symptoms, so its rise space became smaller and its rate of change became slower. In the total sample population, the higher the initial level of depressive symptoms, the faster the development rate of frailty, but the results of subgroup analysis by age and gender showed that the initial level of depressive symptoms was not related to frailty on the development rate. This may be due to the reduced sample size in each group after subgrouping.

The findings of this study were consistent with a number of earlier findings, which indicated that frailty in the middle-aged and older Chinese population predicted the development of depressive symptoms [17, 35]. Meanwhile, frailty was found to be a predictor of depressive symptoms in a cross-sectional research of 576 older persons in an urban community in Shenzhen, China, who were 65 years of age or older [36]. A possible explanation for this relationship was that when older adults suffer from the debilitating syndrome, they had low physical activity, slow movement, fatigue and weakness, and may refuse social activities, and after many times, older adults may develop feelings of uselessness and emptiness, no longer be interested in what was originally interesting, and then depressive symptoms. Another possibility is that interleukin-6 (IL-6) levels are higher in fragile older persons [37], which, as a biomarker of depressive symptoms in older patients, is thought to be more susceptible to geriatric depression. Thus, frailty was a predictor of depressive symptoms.

In the Chinese middle-aged and older adults, a striking finding of this study was that depressive symptoms were a predictor of frailty. This finding was inconsistent with a previous longitudinal study [17], and differences in results may be due to different methods of assessing frailty. However, another cross-sectional study of 5844 participants from seven cities in China showed that depression was a risk factor for frailty [12]. As well, using data from the Rugao Longevity and Aging Study (RuLAS), an analysis of 1168 Chinese older adults aged 70 years and older, Zhang et al. found that depressive symptoms were a risk factor for frailty [38]. In addition, in studies of older adultss from Brazil and Latin America, the results also showed that depression increased the risk of frailty [11, 39]. Therefore, depressive symptoms may also increase the risk of frailty in the older Chinese population. One possible explanation was that depression causes depressed mood, weight loss and loss of appetite in older adults, which in turn triggers symptoms such as reduced mobility, weakness and falls, increasing their risk of developing frailty.

Our study identifies a meaningful relationship with a bidirectional causal association between frailty and depressive symptoms in the Chinese middle-aged and older adults. This is in accordance with the findings of a previous study of older adults in rural China [16]. In addition, a cross-lagged model analysis using data from the CHARLS database also found a bidirectional relationship between loneliness and frailty [40]. While loneliness was measured by a question on the CESD-10 scale in that study, there may be a bidirectional association between frailty and depressive symptoms among Chinese older adults. Additionally, our research revealed that in middle-aged populations, there is a bidirectional causal relationship between frailty and depressive symptoms that is independent of age. This meant that early recognition and intervention that also focuses on depressive symptoms and frailty in middle-aged populations should be considered to delay further deterioration upon entry into old age. At the same time, our study also found that this bidirectional association was independent of gender. Even women are more likely to experience frailty [41] and depressive symptoms [42] than men. This may be because women tend to have lower muscle strength than men, as well as women undertake more family affairs and stronger symptom tolerance. Even so, the bidirectional causal association of frailty leading to depressive symptoms and depressive symptoms leading to frailty in the middle-aged and older Chinese population remains true.

China is currently experiencing deep aging, with a sizable section of the population being older, and the impact of the older’s frailty and depression on society and public health is tremendous. This study confirms the relationship between changes and bidirectional causality between frailty and depressive symptoms, which can provide some reference significance for frailty and depressive symptom intervention.

First, various screening tools such as the frailty (FRAIL) scale, the clinical frailty scale (CFS), and comprehensive care for the elderly (ICOPE) were used to effectively carry out early middle-aged and elderly frailty screening. As well as using the CESD-10 equivalent scale to effectively carry out early middle-aged and elderly depression symptom screening. Secondly, middle-aged and older adultss are encouraged to take vitamin B6 and vitamin B12 supplements (Neuroforte), calcium and vitamin D supplements (calcium salts) [43]. Finally, encourage middle-aged and older people to carry out physical exercise, gradually increase the intensity, according to each person ‘s adaptability to carry out exercise [44].

The following are the study’s advantages: First, this study used a representative sample of Chinese countries, thus the study results can be generalized to the whole country. Secondly, the parallel latent growth model was used to reveal the development trajectory and the interaction between them from a dynamic perspective. Thirdly, this study used cross-lagged path analysis, which can reliably investigate the bidirectional predictive relationship between frailty and depressive symptoms. The present study, however, also has several limitations: Firstly, the depressive symptom assessment in this study was self-reported; Second, in this cohort study, we eliminated missing values and did not use some effective methods for imputation, which may cause some deviations in the results. Third, the model in this study could only identify linear correlations between frailty and depressive symptoms. However, the relationships between variables were subtle, such as the possible “U” -type relationship between frailty and depressive symptoms, could not be investigated based on the model in this study.

In summary, using longitudinal data from the CHARLS database, our results suggested that health care providers should be more aware of this relationship given the existence of longitudinal and reciprocal relationships between frailty and depressive symptoms, and early screening for frailty in the depressed middle-aged and older adults as well as for depressive symptoms in the frailty middle-aged and older adults was also necessary to avoid the coexistence of frailty and depressive symptoms. In addition, we suggested that mental health related measures should also be increased in interventions to improve frailty. Future studies should investigate the relationship between frailty and response to antidepressant medication and whether there are common mechanisms leading to frailty and depressive symptoms.

### Electronic supplementary material

Below is the link to the electronic supplementary material.

**Supplementary Material 1**: Supplementary Table 1. Items and assigned value of frailty index. Supplementary Figure 1. Spearman correlation between frailty and depressive symptoms at T1, T2, T3 and T4 in persons aged 45 - 59 years. Supplementary Figure 2. Spearman correlation between frailty and depressive symptoms at T1, T2, T3 and T4 in persons aged ≥ 60 years. Supplementary Figure 3. Spearman correlation between frailty and depressive symptoms at T1, T2, T3 and T4 in Male. Supplementary Figure 4. Spearman correlation between frailty and depressive symptoms at T1, T2, T3 and T4 in Female. Supplementary Figure 5. A parallel latent growth model for depressive symptoms on frailty in persons aged 45 - 59 years. Supplementary Figure 6. A parallel latent growth model for frailty on depressive symptoms in persons aged 45 - 59 years. Supplementary Figure 7. A parallel latent growth model for depressive symptoms on frailty in persons aged ≥ 60 years. Supplementary Figure 8. A parallel latent growth model for frailty on depressive symptoms in persons aged ≥ 60 years. Supplementary Figure 9. A parallel latent growth model for depressive symptoms on frailty in Male. Supplementary Figure 10. A parallel latent growth model for frailty on depressive symptoms in Male. Supplementary Figure 11. A parallel latent growth model for depressive symptoms on frailty in Female. Supplementary Figure 12. A parallel latent growth model for frailty on depressive symptoms in Female. Supplementary Table 2. Parallel latent growth model adjusted covariate parameters. Supplementary Figure 13. Cross-lagged Model for Frailty and Depressive Symptoms in persons aged 45 - 59 years. Supplementary Figure 14. Cross-lagged Model for Frailty and Depressive Symptoms in persons aged ≥ 60 years. Supplementary Figure 15. Cross-lagged Model for Frailty and Depressive Symptoms in Male. Supplementary Figure 16. Cross-lagged Model for Frailty and Depressive Symptoms in Female.

## Data Availability

Dataset from the China Health and Retirement Longitudinal Study (CHARLS) http://charls.pku.edu.cn/.
