# Supplementary material for: Changes in frailty and depressive symptoms among middle-aged and older Chinese people: a nationwide cohort study
Source: BMC Public Health. 2024 Jan 25;24:301. doi: 10.1186/s12889-024-17824-3 (PMC10811919; doi:10.1186/s12889-024-17824-3)
Supplement: Supplementary file 1 — Supplementary Material 1: Supplementary Table 1. Items and assigned value of frailty index. Supplementary Figure 1. Spearman correlation between frailty and depressive symptoms at T1, T2, T3 and T4 in persons aged 45 - 59 years. Supplementary Figure 2. Spearman correlation between frailty and depressive symptoms at T1, T2, T3 and T4 in persons aged ≥ 60 years. Supplementary Figure 3. Spearman correlation between frailty and depressive symptoms at T1, T2, T3 and T4 in Male. Supplementary Figure 4. Spearman correlation between frailty and depressive symptoms at T1, T2, T3 and T4 in Female. Supplementary Figure 5. A parallel latent growth model for depressive symptoms on frailty in persons aged 45 - 59 years. Supplementary Figure 6. A parallel latent growth model for frailty on depressive symptoms in persons aged 45 - 59 years. Supplementary Figure 7. A parallel latent growth model for depressive symptoms on frailty in persons aged ≥ 60 years. Supplementary Figure 8. A parallel latent growth model for frailty on depressive symptoms in persons aged ≥ 60 years. Supplementary Figure 9. A parallel latent growth model for depressive symptoms on frailty in Male. Supplementary Figure 10. A parallel latent growth model for frailty on depressive symptoms in Male. Supplementary Figure 11. A parallel latent growth model for depressive symptoms on frailty in Female. Supplementary Figure 12. A parallel latent growth model for frailty on depressive symptoms in Female. Supplementary Table 2. Parallel latent growth model adjusted covariate parameters. Supplementary Figure 13. Cross-lagged Model for Frailty and Depressive Symptoms in persons aged 45 - 59 years. Supplementary Figure 14. Cross-lagged Model for Frailty and Depressive Symptoms in persons aged ≥ 60 years. Supplementary Figure 15. Cross-lagged Model for Frailty and Depressive Symptoms in Male. Supplementary Figure 16. Cross-lagged Model for Frailty and Depressive Symptoms in Female. [file 12889_2024_17824_MOESM1_ESM.docx]

**Changes in frailty and depressive symptoms among middle-aged and elderly Chinese: a nationwide cohort study**

**Supplementary materials**

**Contents**

[Supplementary Table 1. Items and assigned value of frailty index. 3](#_Toc1041481532)

[Supplementary Figure 1. Spearman correlation between frailty and depressive symptoms at T1, T2, T3 and T4 in persons aged 45 - 59 years. 6](#_Toc238877949)

[Supplementary Figure 2. Spearman correlation between frailty and depressive symptoms at T1, T2, T3 and T4 in persons aged ≥ 60 years. 7](#_Toc1330223774)

[Supplementary Figure 3. Spearman correlation between frailty and depressive symptoms at T1, T2, T3 and T4 in Male. 8](#_Toc740373041)

[Supplementary Figure 4. Spearman correlation between frailty and depressive symptoms at T1, T2, T3 and T4 in Female. 9](#_Toc459496505)

[Supplementary Figure 5. A parallel latent growth model for depressive symptoms on frailty in persons aged 45 - 59 years. 10](#_Toc406564923)

[Supplementary Figure 6. A parallel latent growth model for frailty on depressive symptoms in persons aged 45 - 59 years. 11](#_Toc1520454277)

[Supplementary Figure 7. A parallel latent growth model for depressive symptoms on frailty in persons aged ≥ 60 years. 12](#_Toc1367117886)

[Supplementary Figure 8. A parallel latent growth model for frailty on depressive symptoms in persons aged ≥ 60 years. 13](#_Toc1222770749)

[Supplementary Figure 9. A parallel latent growth model for depressive symptoms on frailty in Male. 14](#_Toc1836960300)

[Supplementary Figure 10. A parallel latent growth model for frailty on depressive symptoms in Male. 15](#_Toc1651000682)

[Supplementary Figure 11. A parallel latent growth model for depressive symptoms on frailty in Female. 16](#_Toc732259487)

[Supplementary Figure 12. A parallel latent growth model for frailty on depressive symptoms in Female. 17](#_Toc573012192)

[Supplementary Table 2. Parallel latent growth model adjusted covariate parameters. 18](#_Toc1299237796)

[Supplementary Figure 13. Cross-lagged Model for Frailty and Depressive Symptoms in persons aged 45 - 59 years. 23](#_Toc675914676)

[Supplementary Figure 14. Cross-lagged Model for Frailty and Depressive Symptoms in persons aged ≥ 60 years. 24](#_Toc2056950549)

[Supplementary Figure 15. Cross-lagged Model for Frailty and Depressive Symptoms in Male. 25](#_Toc1149615626)

[Supplementary Figure 16. Cross-lagged Model for Frailty and Depressive Symptoms in Female. 26](#_Toc1414894162)

**Supplementary Table 1.** Items and assigned value of frailty index.

|  | **Variable** | **Assigned value** |
| --- | --- | --- |
| Muscle Capability Indicators | Getting up from a chair after sitting for a long period | No difficulty=0; A little difficulty=0.33; Need help=0.67; Unable to complete=1 |
|  | Stooping, kneeling, or crouching | No difficulty=0; A little difficulty=0.33; Need help=0.67; Unable to complete=1 |
|  | Reaching or extending your arms above shoulder level | No difficulty=0; A little difficulty=0.33; Need help=0.67; Unable to complete=1 |
|  | Lifting or carrying weights over 5 kg, like a heavy bag of groceries | No difficulty=0; A little difficulty=0.33; Need help=0.67; Unable to complete=1 |
|  | Picking up a small coin from a table | No difficulty=0; A little difficulty=0.33; Need help=0.67; Unable to complete=1 |
| Mobility Indicators | running or jogging about 1 km | No difficulty=0; A little difficulty=0.33; Need help=0.67; Unable to complete=1 |
|  | Walking 1 km | No difficulty=0; A little difficulty=0.33; Need help=0.67; Unable to complete=1 |
|  | Climbing several flights of stairs without resting | No difficulty=0; A little difficulty=0.33; Need help=0.67; Unable to complete=1 |
| Instrumental Activities of Daily Living Scale | doing household chores | No difficulty=0; A little difficulty=0.33; Need help=0.67; Unable to complete=1 |
|  | preparing hot meals | No difficulty=0; A little difficulty=0.33; Need help=0.67; Unable to complete=1 |
|  | shopping for groceries | No difficulty=0; A little difficulty=0.33; Need help=0.67; Unable to complete=1 |
|  | managing your money | No difficulty=0; A little difficulty=0.33; Need help=0.67; Unable to complete=1 |
|  | taking medications | No difficulty=0; A little difficulty=0.33; Need help=0.67; Unable to complete=1 |
| Basic Activity of Daily Living | dressing | No difficulty=0; A little difficulty=0.33; Need help=0.67; Unable to complete=1 |
|  | bathing or showering | No difficulty=0; A little difficulty=0.33; Need help=0.67; Unable to complete=1 |
|  | eating | No difficulty=0; A little difficulty=0.33; Need help=0.67; Unable to complete=1 |
|  | getting into or out of bed | No difficulty=0; A little difficulty=0.33; Need help=0.67; Unable to complete=1 |
|  | using the toilet, including getting up and down | No difficulty=0; A little difficulty=0.33; Need help=0.67; Unable to complete=1 |
|  | controlling urination and defecation | No difficulty=0; A little difficulty=0.33; Need help=0.67; Unable to complete=1 |
| Self-assessed health status | self-rated health | Excellent=0; Very good=0.25; Good=0.5; Fair=0.75; Poor=1 |
| Chronic disease | Hypertension | No=0; Yes=1 |
|  | Dyslipidemia | No=0; Yes=1 |
|  | Diabetes or high blood sugar | No=0; Yes=1 |
|  | Cancer or malignant tumor | No=0; Yes=1 |
|  | Chronic lung diseases | No=0; Yes=1 |
|  | Liver disease | No=0; Yes=1 |
|  | Heart attack | No=0; Yes=1 |
|  | Stroke | No=0; Yes=1 |
|  | Kidney disease | No=0; Yes=1 |
|  | Stomach or other digestive disease | No=0; Yes=1 |
|  | Emotional, nervous, or psychiatric problems | No=0; Yes=1 |
|  | Memory-related disease | No=0; Yes=1 |
|  | Arthritis or rheumatism | No=0; Yes=1 |
|  | Asthma | No=0; Yes=1 |
|  | Physical disabilities | No=0; Yes=1 |
|  | Brain damage/mental retardation | No=0; Yes=1 |
|  | Vision problem | No=0; Yes=1 |
|  | Hearing problem | No=0; Yes=1 |
|  | Speech impediment | No=0; Yes=1 |
| Disabilities | Physical disabilities | No=0; Yes=1 |
|  | Brain damage/mental retardation | No=0; Yes=1 |
|  | Vision problem | No=0; Yes=1 |
|  | Hearing problem | No=0; Yes=1 |
|  | Speech impediment | No=0; Yes=1 |
| Cognitive function | TICS-m | TICS-m total score/21 |

**
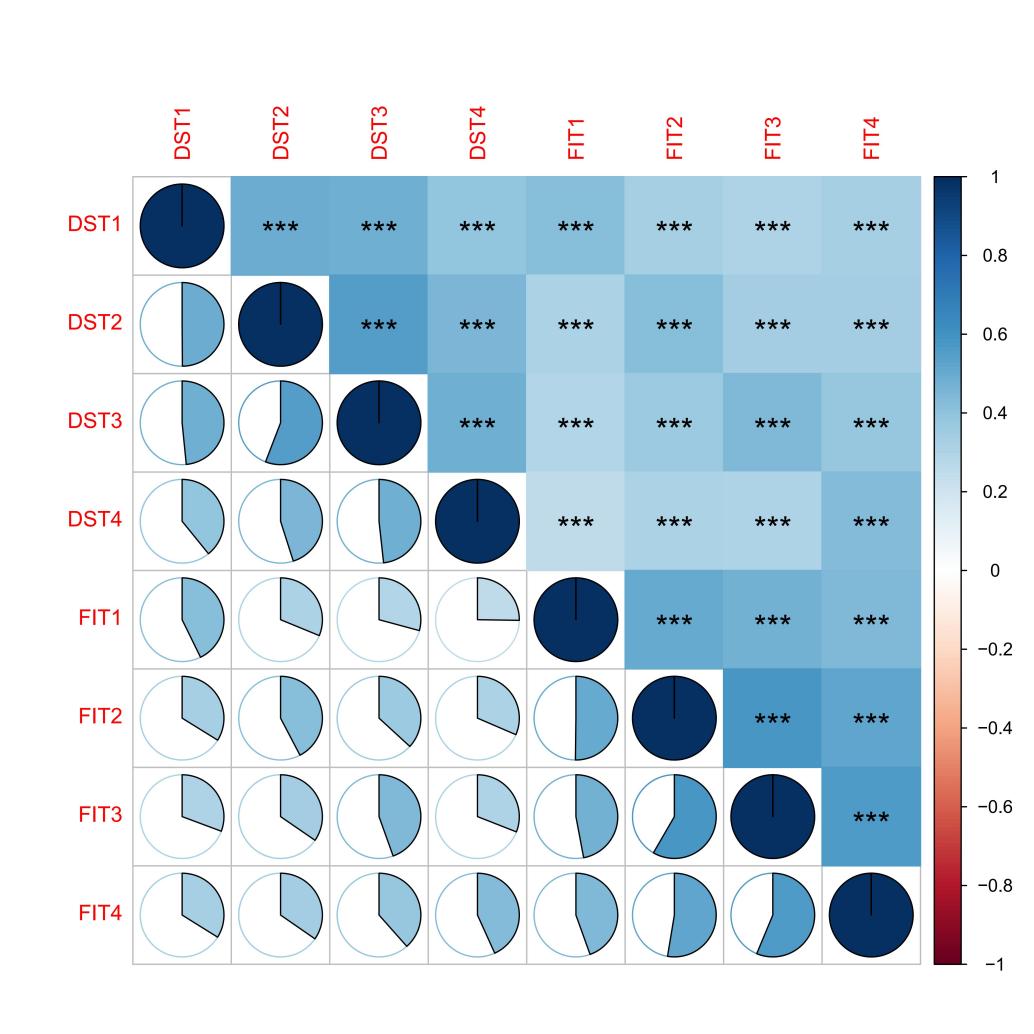
**

**Supplementary Figure 1.** Spearman correlation between frailty and depressive symptoms at T1, T2, T3 and T4 in persons aged 45 - 59 years.

Note: FIT1 = frailty index in 2011; FIT2 = frailty index in 2013; FIT3 = frailty index in 2015; FIT4 = frailty index in 2018; DST1 = depressive symptoms in 2011; DST2 = depressive symptoms in 2013; DST3 = depressive symptoms in 2015; DST4 = depressive symptoms in 2018;

***: *P*<0.001;

**
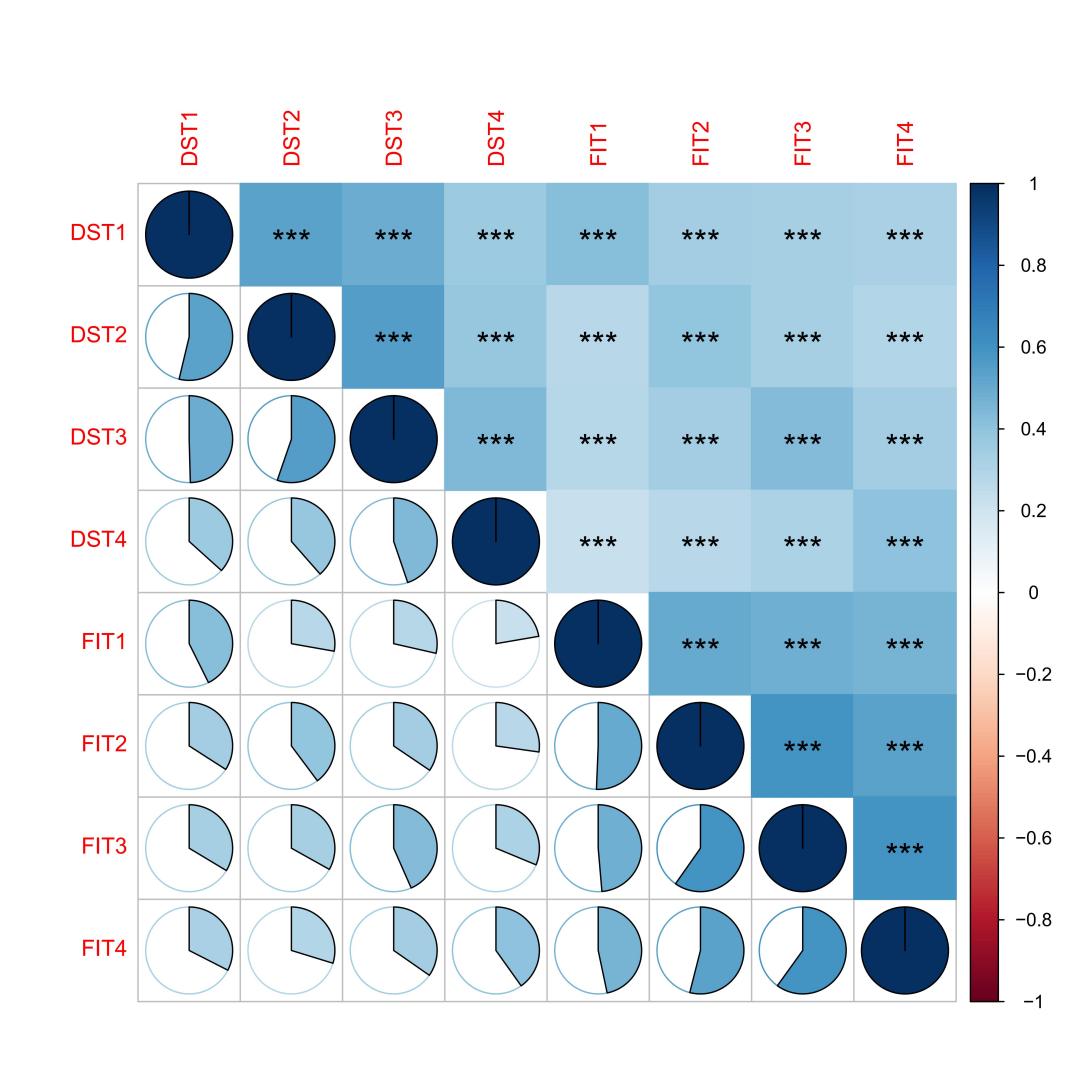
**

**Supplementary Figure 2.** Spearman correlation between frailty and depressive symptoms at T1, T2, T3 and T4 in persons aged ≥ 60 years.

Note: FIT1 = frailty index in 2011; FIT2 = frailty index in 2013; FIT3 = frailty index in 2015; FIT4 = frailty index in 2018; DST1 = depressive symptoms in 2011; DST2 = depressive symptoms in 2013; DST3 = depressive symptoms in 2015; DST4 = depressive symptoms in 2018;

***:P<0.001;

**
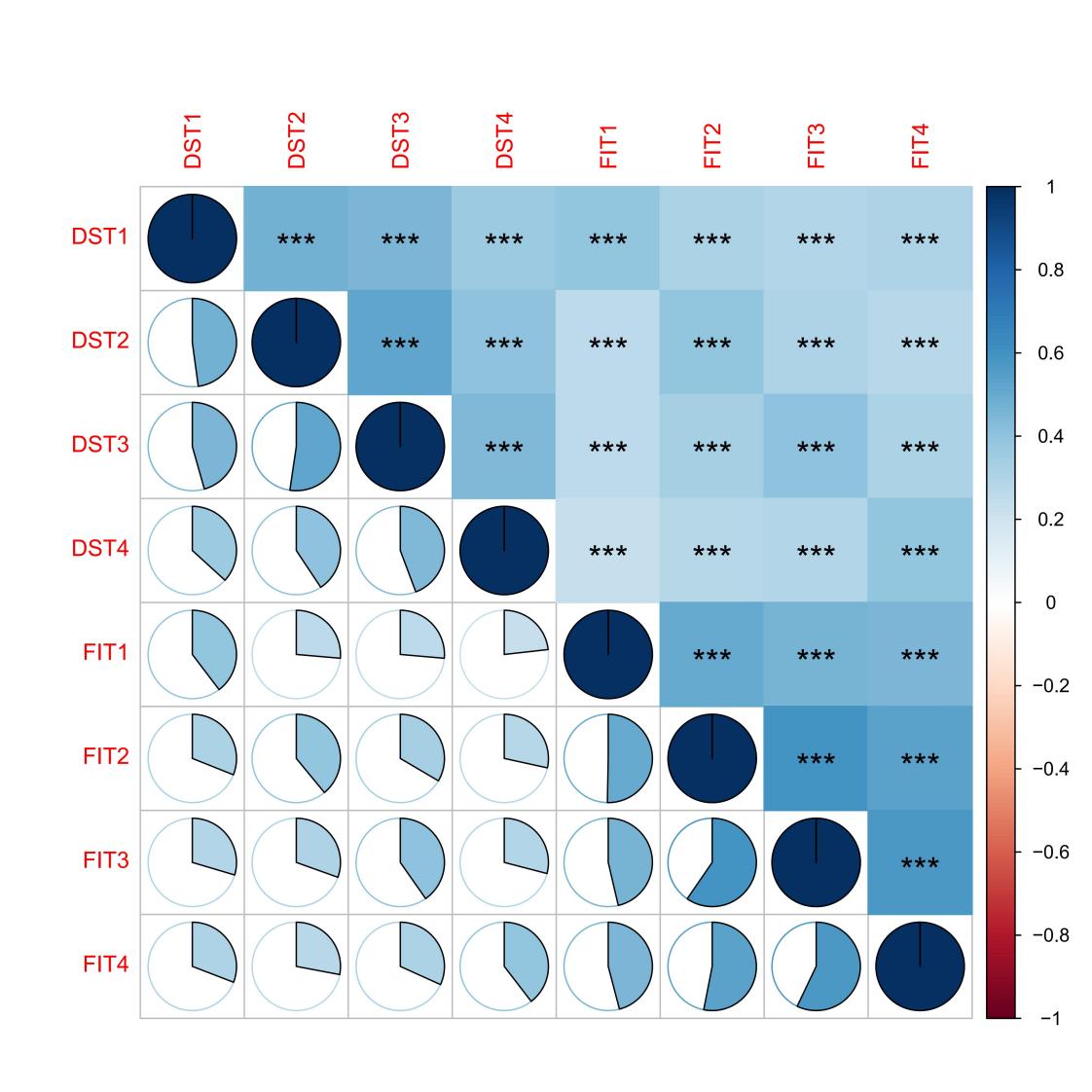
**

**Supplementary Figure 3.** Spearman correlation between frailty and depressive symptoms at T1, T2, T3 and T4 in Male.

Note: FIT1 = frailty index in 2011; FIT2 = frailty index in 2013; FIT3 = frailty index in 2015; FIT4 = frailty index in 2018; DST1 = depressive symptoms in 2011; DST2 = depressive symptoms in 2013; DST3 = depressive symptoms in 2015; DST4 = depressive symptoms in 2018;

***:P<0.001;

**
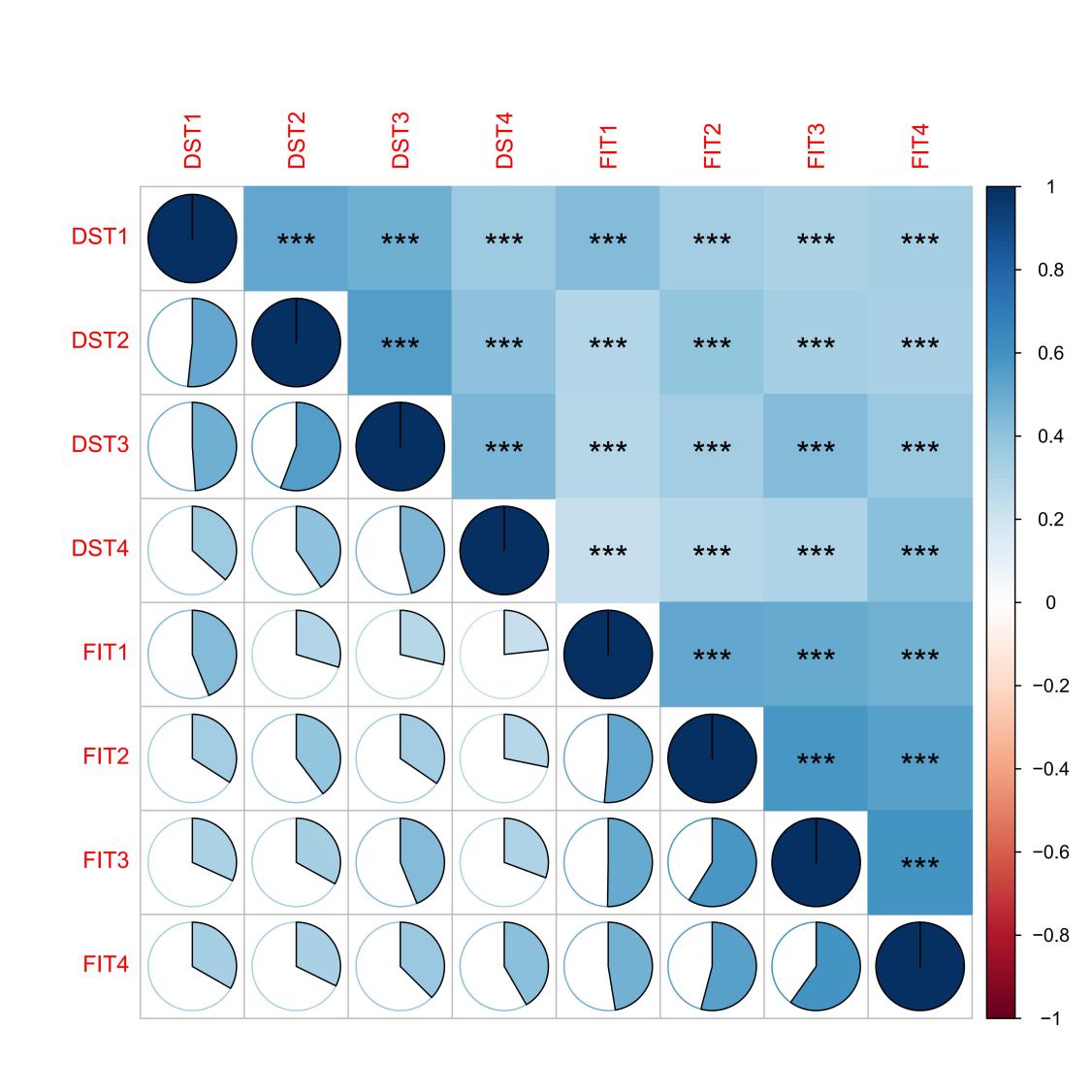
**

**Supplementary Figure 4.** Spearman correlation between frailty and depressive symptoms at T1, T2, T3 and T4 in Female.

Note: FIT1 = frailty index in 2011; FIT2 = frailty index in 2013; FIT3 = frailty index in 2015; FIT4 = frailty index in 2018; DST1 = depressive symptoms in 2011; DST2 = depressive symptoms in 2013; DST3 = depressive symptoms in 2015; DST4 = depressive symptoms in 2018;

***:P<0.001;


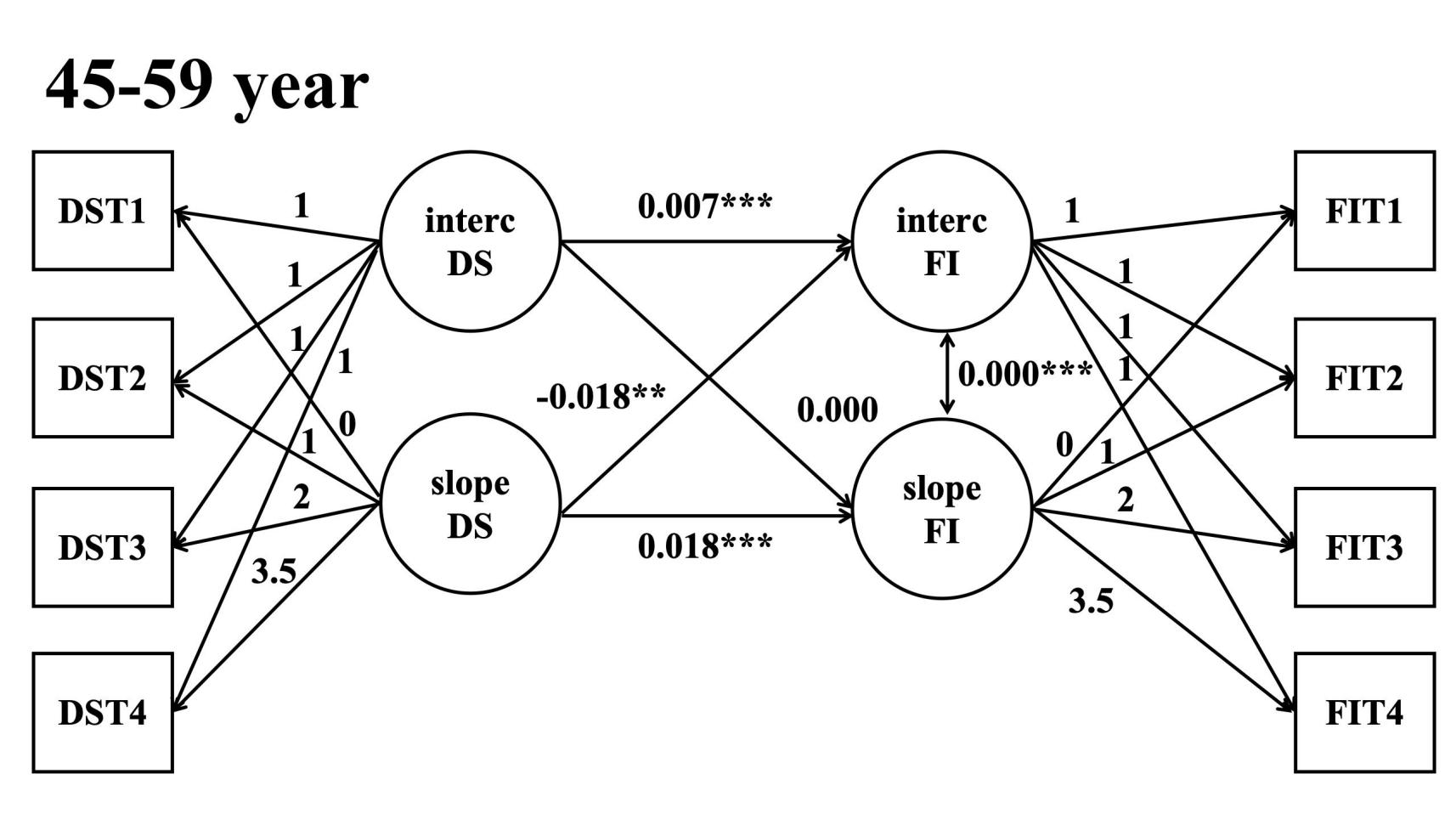


**Supplementary Figure 5.** A parallel latent growth model for depressive symptoms on frailty in persons aged 45 - 59 years.

Note: DS = depressive symptoms; FI = frailty index; FIT1 = frailty index in 2011; FIT2 = frailty index in 2013; FIT3 = frailty index in 2015; FIT4 = frailty index in 2018; DST1 = depressive symptoms in 2011; DST2 = depressive symptoms in 2013; DST3 = depressive symptoms in 2015; DST4 = depressive symptoms in 2018; Adjusting covariates: Education; Marital status; Smoking; Drinking; Gender;

***: P<0.001; **: P<0.01; *: P<0.05.

**
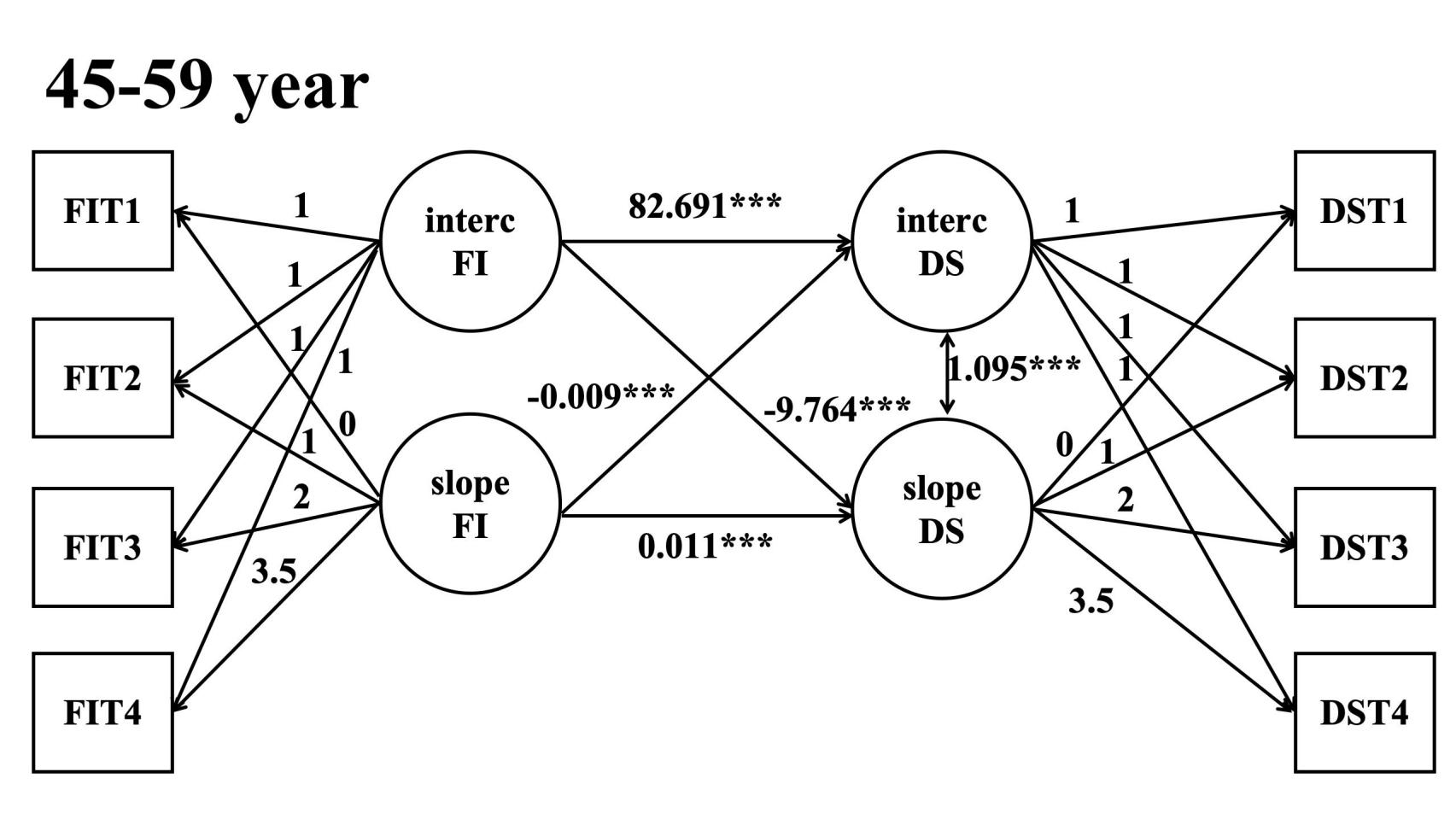
**

**Supplementary Figure 6.** A parallel latent growth model for frailty on depressive symptoms in persons aged 45 - 59 years.

Note: DS = depressive symptoms; FI = frailty index; FIT1 = frailty index in 2011; FIT2 = frailty index in 2013; FIT3 = frailty index in 2015; FIT4 = frailty index in 2018; DST1 = depressive symptoms in 2011; DST2 = depressive symptoms in 2013; DST3 = depressive symptoms in 2015; DST4 = depressive symptoms in 2018; Adjusting covariates: Education; Marital status; Smoking; Drinking; Gender;

***: P<0.001; **: P<0.01; *: P<0.05.

**
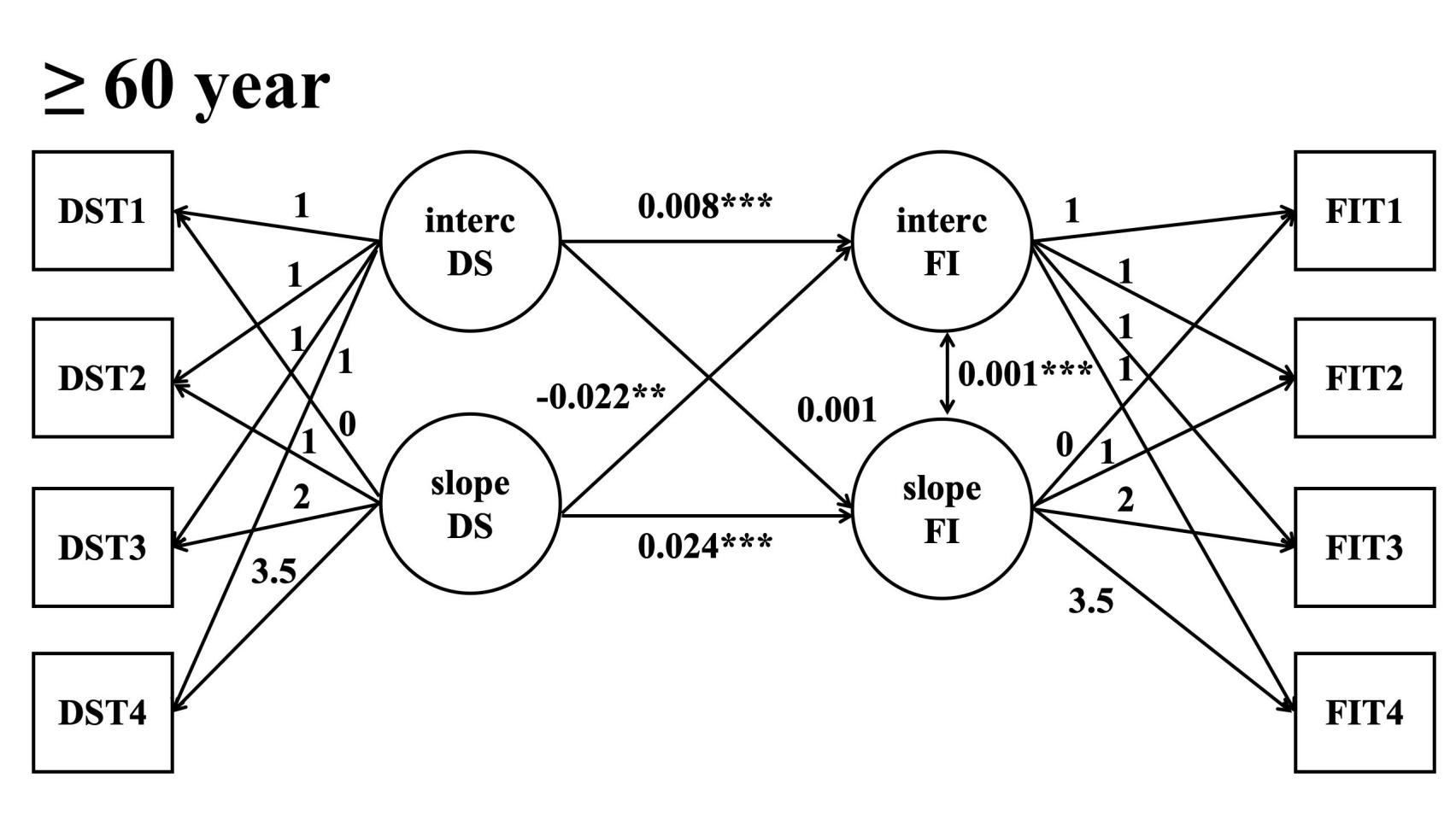
**

**Supplementary Figure 7.** A parallel latent growth model for depressive symptoms on frailty in persons aged ≥ 60 years.

Note: DS = depressive symptoms; FI = frailty index; FIT1 = frailty index in 2011; FIT2 = frailty index in 2013; FIT3 = frailty index in 2015; FIT4 = frailty index in 2018; DST1 = depressive symptoms in 2011; DST2 = depressive symptoms in 2013; DST3 = depressive symptoms in 2015; DST4 = depressive symptoms in 2018; Adjusting covariates: Education; Marital status; Smoking; Drinking; Gender;

***: P<0.001; **: P<0.01; *: P<0.05.

**
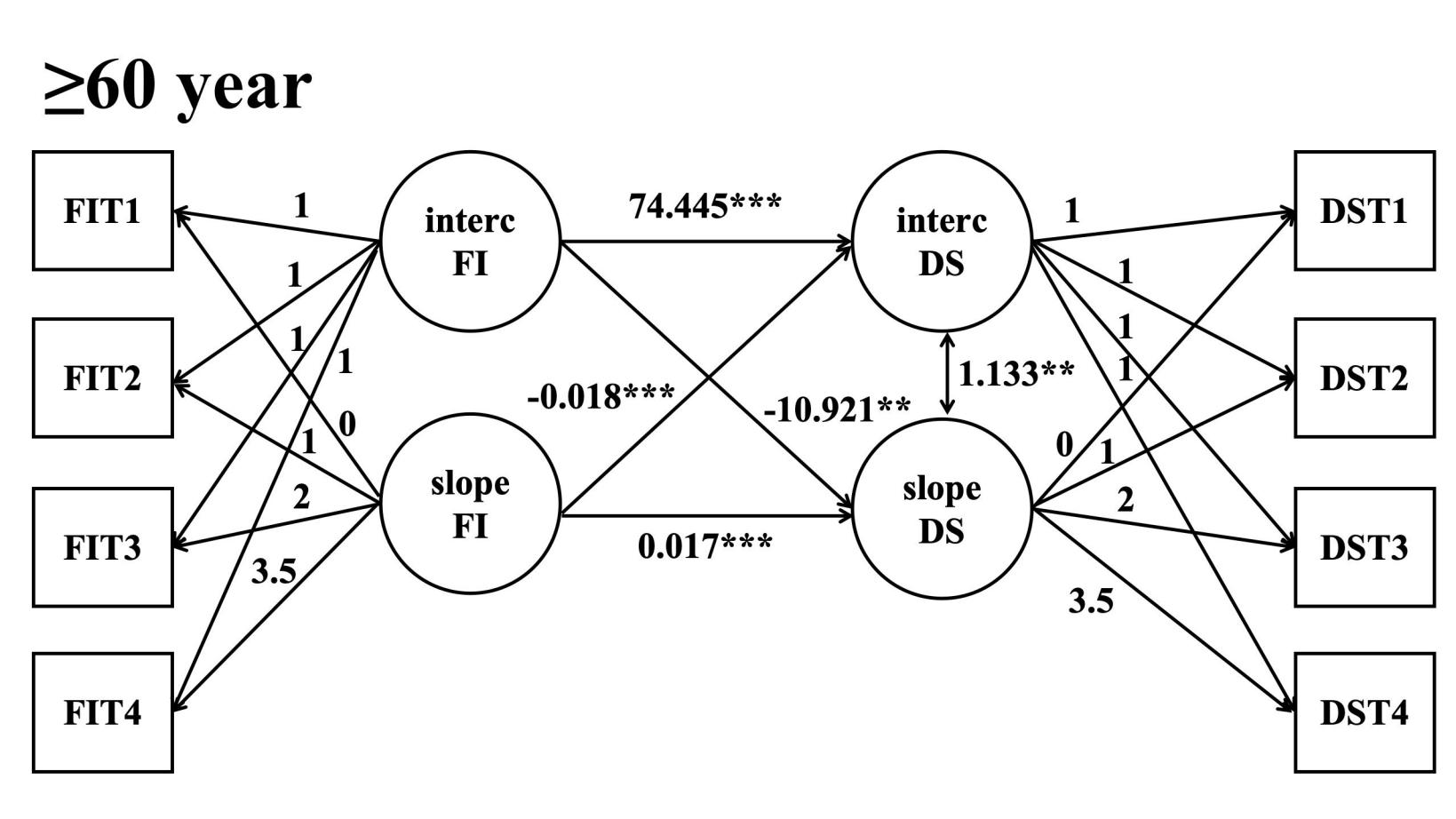
**

**Supplementary Figure 8.** A parallel latent growth model for frailty on depressive symptoms in persons aged ≥ 60 years.

Note: DS = depressive symptoms; FI = frailty index; FIT1 = frailty index in 2011; FIT2 = frailty index in 2013; FIT3 = frailty index in 2015; FIT4 = frailty index in 2018; DST1 = depressive symptoms in 2011; DST2 = depressive symptoms in 2013; DST3 = depressive symptoms in 2015; DST4 = depressive symptoms in 2018; Adjusting covariates: Education; Marital status; Smoking; Drinking; Gender;

***: P<0.001; **: P<0.01; *: P<0.05.

**
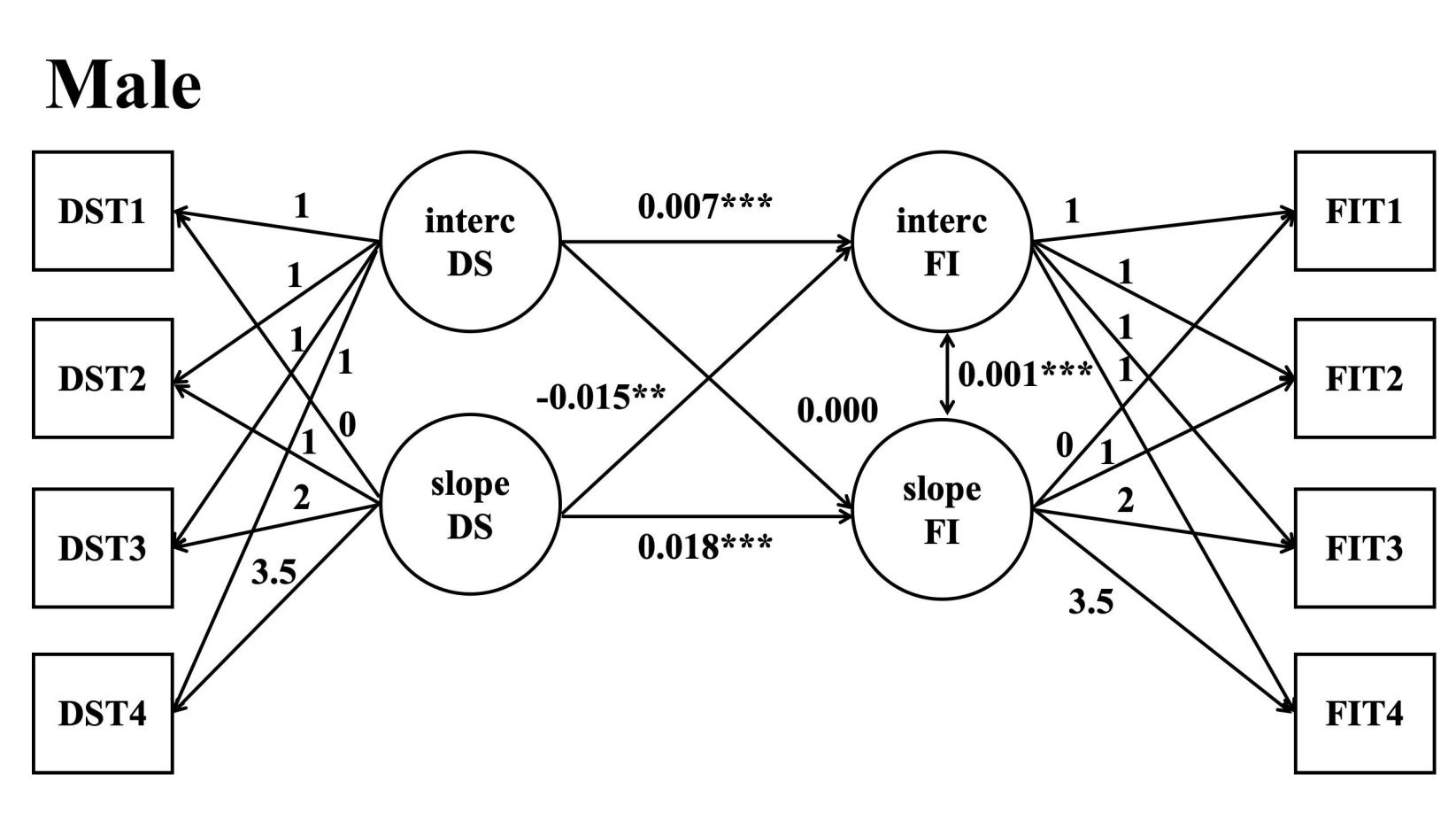
**

**Supplementary Figure 9.** A parallel latent growth model for depressive symptoms on frailty in Male.

Note: DS = depressive symptoms; FI = frailty index; FIT1 = frailty index in 2011; FIT2 = frailty index in 2013; FIT3 = frailty index in 2015; FIT4 = frailty index in 2018; DST1 = depressive symptoms in 2011; DST2 = depressive symptoms in 2013; DST3 = depressive symptoms in 2015; DST4 = depressive symptoms in 2018; Adjusting covariates: Age; Education; Marital status; Smoking; Drinking;

**
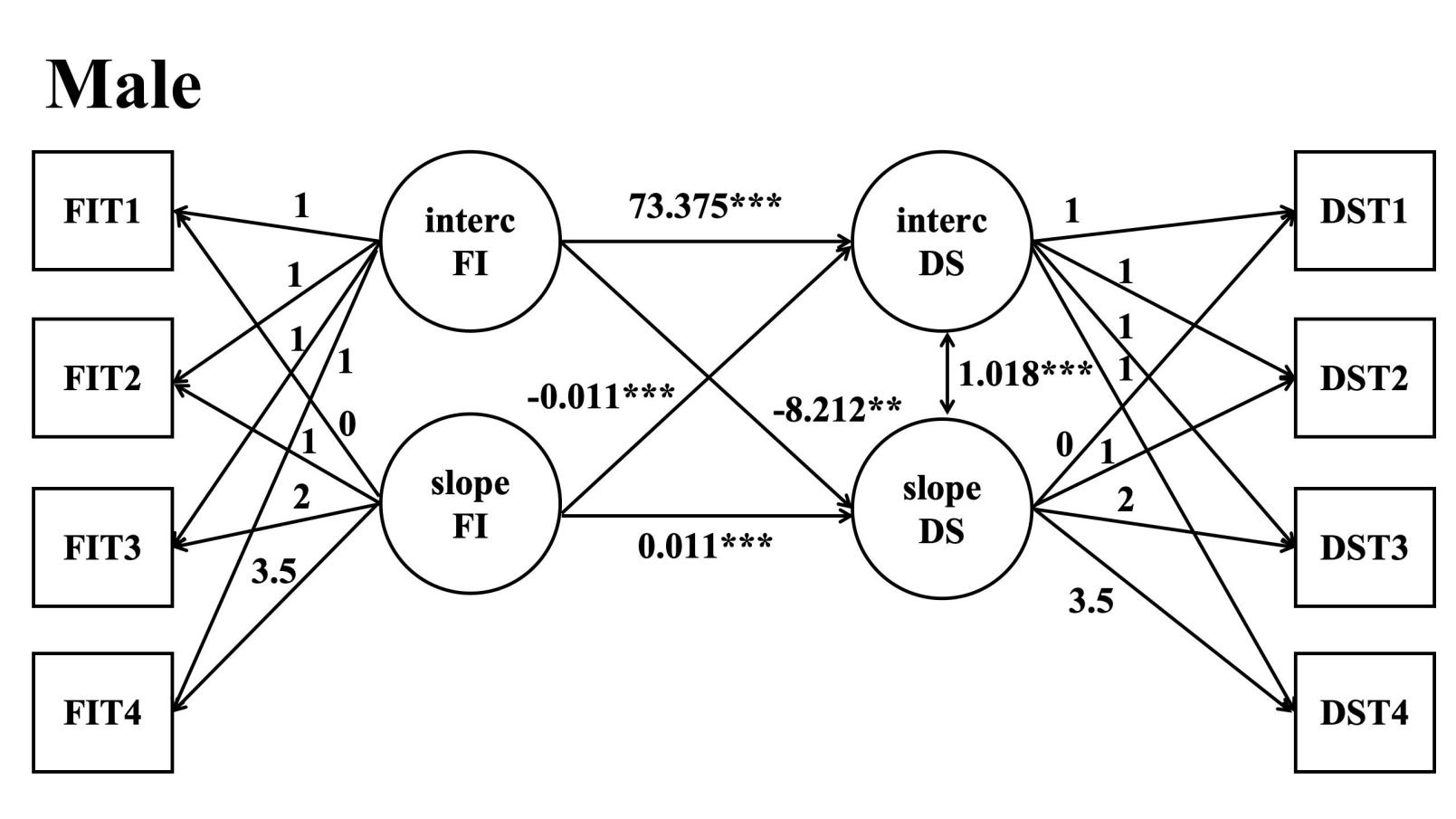
**

**Supplementary Figure 10.** A parallel latent growth model for frailty on depressive symptoms in Male.

Note: DS = depressive symptoms; FI = frailty index; FIT1 = frailty index in 2011; FIT2 = frailty index in 2013; FIT3 = frailty index in 2015; FIT4 = frailty index in 2018; DST1 = depressive symptoms in 2011; DST2 = depressive symptoms in 2013; DST3 = depressive symptoms in 2015; DST4 = depressive symptoms in 2018; Adjusting covariates: Age; Education; Marital status; Smoking; Drinking;

***: P<0.001; **: P<0.01; *: P<0.05.

**
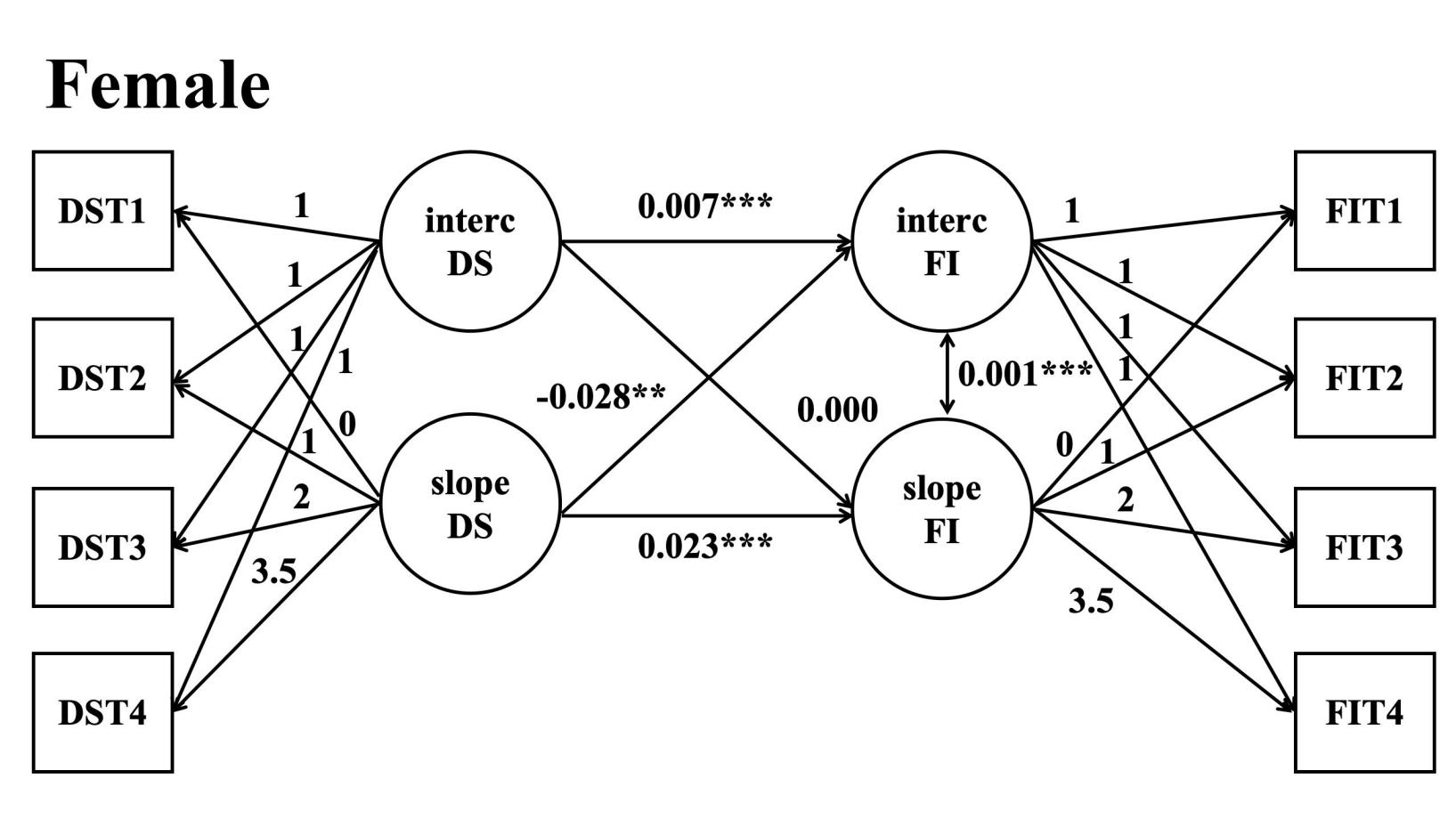
**

**Supplementary Figure 11.** A parallel latent growth model for depressive symptoms on frailty in Female.

Note: DS = depressive symptoms; FI = frailty index; FIT1 = frailty index in 2011; FIT2 = frailty index in 2013; FIT3 = frailty index in 2015; FIT4 = frailty index in 2018; DST1 = depressive symptoms in 2011; DST2 = depressive symptoms in 2013; DST3 = depressive symptoms in 2015; DST4 = depressive symptoms in 2018; Adjusting covariates: Age; Education; Marital status; Smoking; Drinking;

**
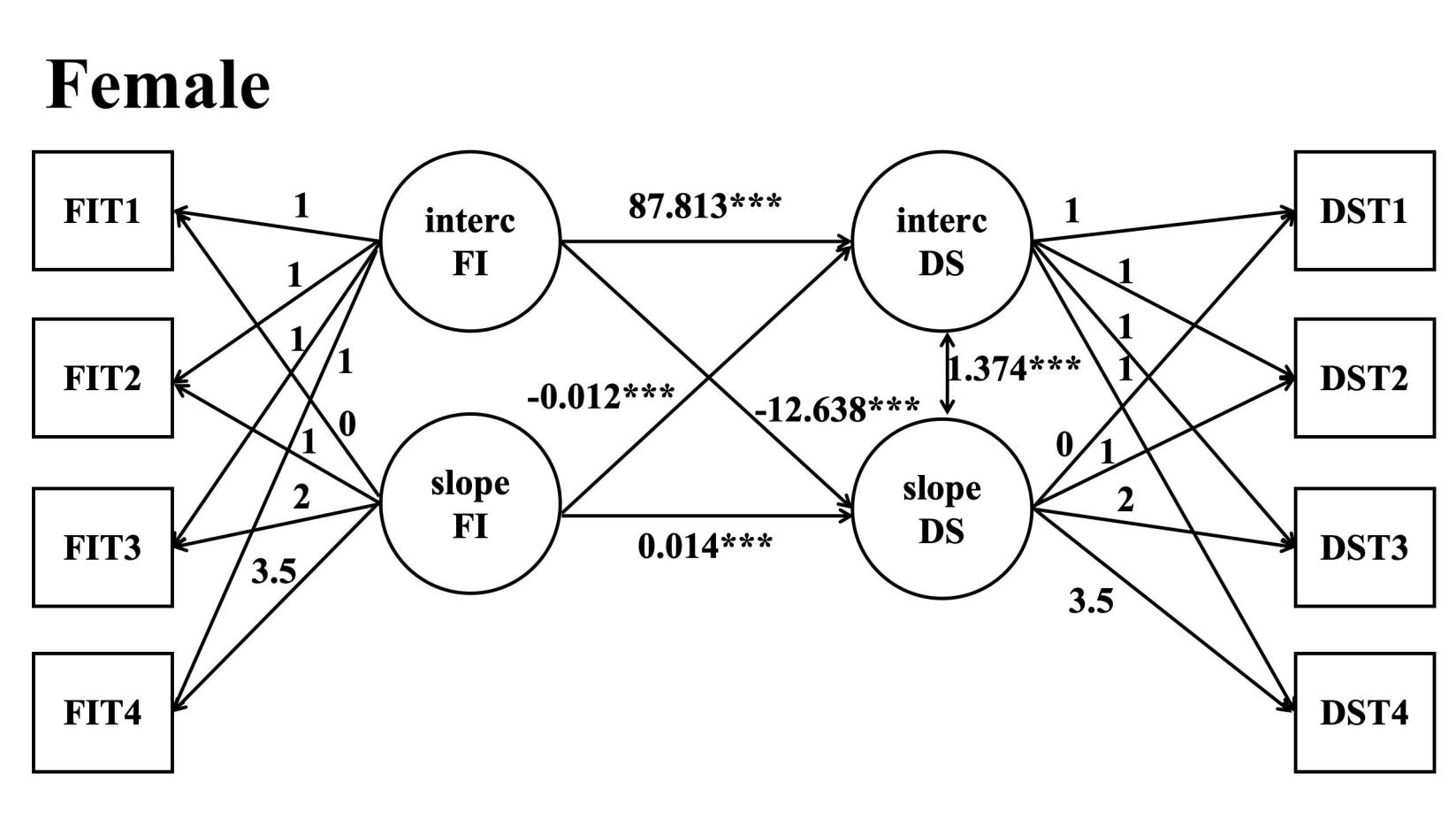
**

**Supplementary Figure 12.** A parallel latent growth model for frailty on depressive symptoms in Female.

Note: DS = depressive symptoms; FI = frailty index; FIT1 = frailty index in 2011; FIT2 = frailty index in 2013; FIT3 = frailty index in 2015; FIT4 = frailty index in 2018; DST1 = depressive symptoms in 2011; DST2 = depressive symptoms in 2013; DST3 = depressive symptoms in 2015; DST4 = depressive symptoms in 2018; Adjusting covariates: Age; Education; Marital status; Smoking; Drinking;

***: P<0.001; **: P<0.01; *: P<0.05.

**Supplementary Table 2.** Parallel latent growth model adjusted covariate parameters.

|  |  | Intercept D | | | Slope D | | | Intercept F | | | Slope F | | |
| --- | --- | --- | --- | --- | --- | --- | --- | --- | --- | --- | --- | --- | --- |
|  |  | *β* | *se* | *P* | *β* | *se* | *P* | *β* | *se* | *P* | *β* | *se* | *P* |
| Total |  |  |  |  |  |  |  |  |  |  |  |  |  |
| Depressive symptoms on Frailty | Age | 0.001 | 0.008 | 0.880 | 0.004 | 0.003 | 0.173 | 0.001 | 0.001 | <0.001 | 0.001 | 0.001 | 0.019 |
|  | Education | -0.460 | 0.034 | <0.001 | -0.061 | 0.013 | <0.001 | 0.001 | 0.001 | 0.972 | 0.001 | 0.001 | 0.051 |
|  | Marital status | 0.366 | 0.061 | <0.001 | -0.050 | 0.025 | 0.044 | -0.003 | 0.001 | 0.003 | 0.001 | 0.001 | 0.025 |
|  | Smoking | -0.548 | 0.167 | 0.001 | -0.036 | 0.066 | 0.583 | 0.001 | 0.002 | 0.941 | 0.001 | 0.001 | 0.925 |
|  | Drinking | 0.148 | 0.079 | 0.061 | 0.042 | 0.032 | 0.178 | 0.005 | 0.001 | <0.001 | -0.001 | 0.001 | 0.038 |
|  | Gender | 1.497 | 0.181 | <0.001 | 0.134 | 0.072 | 0.062 | 0.003 | 0.003 | 0.196 | 0.001 | 0.002 | 0.754 |
| Frailty on Depressive symptoms | Age | -0.084 | 0.009 | <0.001 | 0.016 | 0.003 | <0.001 | 0.001 | 0.001 | <0.001 | 0.001 | 0.001 | <0.001 |
|  | Education | -0.295 | 0.034 | <0.001 | -0.083 | 0.014 | <0.001 | -0.002 | 0.001 | <0.001 | -0.001 | 0.001 | <0.001 |
|  | Marital status | 0.292 | 0.060 | <0.001 | -0.041 | 0.025 | 0.107 | 0.001 | 0.001 | 0.120 | 0.001 | 0.001 | 0.703 |
|  | Smoking | -0.273 | 0.165 | 0.098 | -0.072 | 0.067 | 0.281 | -0.003 | 0.002 | 0.050 | -0.001 | 0.001 | 0.234 |
|  | Drinking | -0.262 | 0.079 | 0.001 | 0.096 | 0.032 | 0.003 | 0.005 | 0.001 | <0.001 | -0.001 | 0.001 | 0.075 |
|  | Gender | 0.579 | 0.183 | 0.002 | 0.254 | 0.074 | 0.001 | 0.011 | 0.002 | <0.001 | 0.003 | 0.001 | <0.001 |
| 45-59 year |  |  |  |  |  |  |  |  |  |  |  |  |  |
| Depressive symptoms on Frailty | Education | -0.480 | 0.042 | <0.001 | -0.053 | 0.017 | 0.001 | 0.000 | 0.001 | 0.435 | 0.000 | 0.000 | 0.468 |
|  | Marital status | 0.720 | 0.093 | <0.001 | -0.087 | 0.034 | 0.011 | -0.003 | 0.001 | 0.019 | 0.002 | 0.001 | 0.033 |
|  | Smoking | -0.443 | 0.218 | 0.043 | -0.055 | 0.083 | 0.508 | -0.001 | 0.003 | 0.845 | 0.000 | 0.002 | 0.978 |
|  | Drinking | 0.234 | 0.099 | 0.019 | 0.010 | 0.038 | 0.795 | 0.002 | 0.001 | 0.110 | 0.000 | 0.001 | 0.523 |
|  | Gender | 1.429 | 0.236 | <0.001 | 0.172 | 0.090 | 0.057 | 0.002 | 0.00 | 0.595 | -0.001 | 0.002 | 0.647 |
| Frailty on Depressive symptoms | Education | -0.248 | 0.043 | <0.001 | -0.080 | 0.017 | <0.001 | -0.003 | 0.000 | <0.001 | -0.001 | 0.000 | <0.001 |
|  | Marital status | 0.435 | 0.095 | <0.001 | -0.054 | 0.035 | 0.127 | 0.003 | 0.001 | 0.001 | 0.000 | 0.000 | 0.472 |
|  | Smoking | -0.223 | 0.221 | 0.312 | -0.081 | 0.085 | 0.339 | -0.003 | 0.002 | 0.226 | -0.001 | 0.001 | 0.218 |
|  | Drinking | -0.056 | 0.101 | 0.578 | 0.044 | 0.039 | 0.255 | 0.004 | 0.001 | <0.001 | 0.000 | 0.000 | 0.396 |
|  | Gender | 0.730 | 0.246 | 0.003 | 0.254 | 0.094 | 0.007 | 0.008 | 0.002 | 0.001 | 0.002 | 0.001 | 0.017 |
| ≥ 60 year |  |  |  |  |  |  |  |  |  |  |  |  |  |
| Depressive symptoms on Frailty | Education | -0.404 | 0.055 | <0.001 | -0.081 | 0.022 | <0.001 | -0.001 | 0.001 | 0.555 | 0.001 | 0.001 | 0.098 |
|  | Marital status | 0.149 | 0.077 | 0.054 | -0.029 | 0.033 | 0.389 | -0.002 | 0.001 | 0.141 | 0.001 | 0.001 | 0.143 |
|  | Smoking | -0.684 | 0.261 | 0.009 | -0.012 | 0.106 | 0.909 | 0.001 | 0.004 | 0.854 | 0.000 | 0.002 | 0.914 |
|  | Drinking | -0.012 | 0.128 | 0.924 | 0.097 | 0.054 | 0.070 | 0.010 | 0.002 | <0.001 | -0.003 | 0.001 | 0.037 |
|  | Gender | 0.002 | 0.003 | 0.474 | 0.061 | 0.115 | 0.592 | 0.003 | 0.004 | 0.535 | 0.002 | 0.003 | 0.474 |
| Frailty on Depressive symptoms | Education | -0.263 | 0.056 | <0.001 | -0.101 | 0.023 | <0.001 | -0.002 | 0.001 | 0.002 | -0.001 | 0.000 | 0.002 |
|  | Marital status | 0.154 | 0.077 | 0.044 | -0.029 | 0.033 | 0.381 | 0.000 | 0.001 | 0.933 | 0.000 | 0.000 | 0.135 |
|  | Smoking | -0.365 | 0.248 | 0.141 | -0.059 | 0.107 | 0.584 | -0.004 | 0.003 | 0.136 | 0.000 | 0.001 | 0.683 |
|  | Drinking | -0.613 | 0.125 | <0.001 | 0.186 | 0.054 | 0.001 | 0.008 | 0.001 | <0.001 | -0.001 | 0.001 | 0.223 |
|  | Gender | 0.629 | 0.277 | 0.023 | 0.215 | 0.119 | 0.070 | 0.014 | 0.003 | <0.001 | 0.003 | 0.001 | 0.011 |
| Male |  |  |  |  |  |  |  |  |  |  |  |  |  |
| Depressive symptoms on Frailty | Age | 0.011 | 0.010 | 0.275 | 0.004 | 0.004 | 0.327 | 0.001 | 0.000 | <0.001 | 0.000 | 0.000 | 0.035 |
|  | Education | -0.394 | 0.047 | <0.001 | -0.055 | 0.020 | 0.005 | 0.000 | 0.001 | 0.897 | 0.001 | 0.000 | 0.184 |
|  | Marital status | 0.507 | 0.089 | <0.001 | -0.075 | 0.037 | 0.040 | -0.004 | 0.001 | 0.004 | 0.002 | 0.001 | 0.013 |
|  | Smoking | -0.560 | 0.181 | 0.002 | -0.019 | 0.073 | 0.789 | 0.005 | 0.002 | 0.057 | -0.001 | 0.001 | 0.303 |
|  | Drinking | 0.361 | 0.085 | <0.001 | 0.025 | 0.035 | 0.477 | 0.003 | 0.001 | 0.006 | -0.001 | 0.001 | 0.159 |
| Frailty on Depressive symptoms | Age | -0.048 | 0.011 | <0.001 | 0.011 | 0.004 | 0.015 | 0.001 | 0.000 | <0.001 | 0.000 | 0.000 | <0.001 |
|  | Education | -0.250 | 0.048 | <0.001 | -0.071 | 0.020 | <0.001 | -0.002 | 0.000 | <0.001 | 0.000 | 0.000 | 0.072 |
|  | Marital status | 0.430 | 0.089 | <0.001 | -0.066 | 0.037 | 0.075 | 0.001 | 0.001 | 0.311 | 0.000 | 0.000 | 0.354 |
|  | Smoking | -0.620 | 0.178 | 0.001 | -0.013 | 0.073 | 0.859 | 0.001 | 0.002 | 0.667 | -0/002 | 0.001 | 0.025 |
|  | Drinking | -0.032 | 0.087 | 0.713 | 0.069 | 0.036 | 0.053 | 0.005 | 0.001 | <0.001 | -0.001 | 0.000 | 0.088 |
| Female |  |  |  |  |  |  |  |  |  |  |  |  |  |
| Depressive symptoms on Frailty | Age | 0.004 | 0.013 | 0.761 | -0.010 | 0.004 | 0.016 | 0.001 | 0.000 | <0.001 | 0.000 | 0.000 | <0.001 |
|  | Education | -0.516 | 0.049 | <0.001 | -0.036 | 0.017 | 0.030 | 0.001 | 0.001 | 0.369 | 0.000 | 0.000 | 0.929 |
|  | Marital status | 0.311 | 0.092 | 0.001 | -0.054 | 0.029 | 0.058 | -0.003 | 0.001 | 0.010 | 0.001 | 0.001 | 0.143 |
|  | Smoking | -0.583 | 0.404 | 0.148 | -0.098 | 0.126 | 0.434 | -0.011 | 0.005 | 0.033 | 0.003 | 0.003 | 0.232 |
|  | Drinking | -0.532 | 0.184 | 0.004 | 0.120 | 0.058 | 0.037 | 0.012 | 0.003 | <0.001 | -0.003 | 0.001 | 0.030 |
| Frailty on Depressive symptoms | Age | -0.103 | 0.014 | <0.001 | 0.005 | 0.005 | 0.301 | 0.001 | 0.000 | <0.001 | 0.000 | 0.000 | <0.001 |
|  | Education | -0.351 | 0.050 | <0.001 | -0.060 | 0.017 | 0.001 | -0.002 | 0.000 | <0.001 | -0.001 | 0.000 | <0.001 |
|  | Marital status | 0.290 | 0.089 | 0.001 | -0.051 | 0.029 | 0.078 | 0.000 | 0.001 | 0.744 | 0.000 | 0.000 | 0.476 |
|  | Smoking | 0.536 | 0.373 | 0.151 | -0.259 | 0.126 | 0.040 | -0.013 | 0.004 | 0.001 | 0.001 | 0.001 | 0.428 |
|  | Drinking | -0.963 | 0.176 | <0.001 | 0.183 | 0.059 | 0.002 | 0.005 | 0.002 | 0.004 | 0.000 | 0.001 | 0.782 |


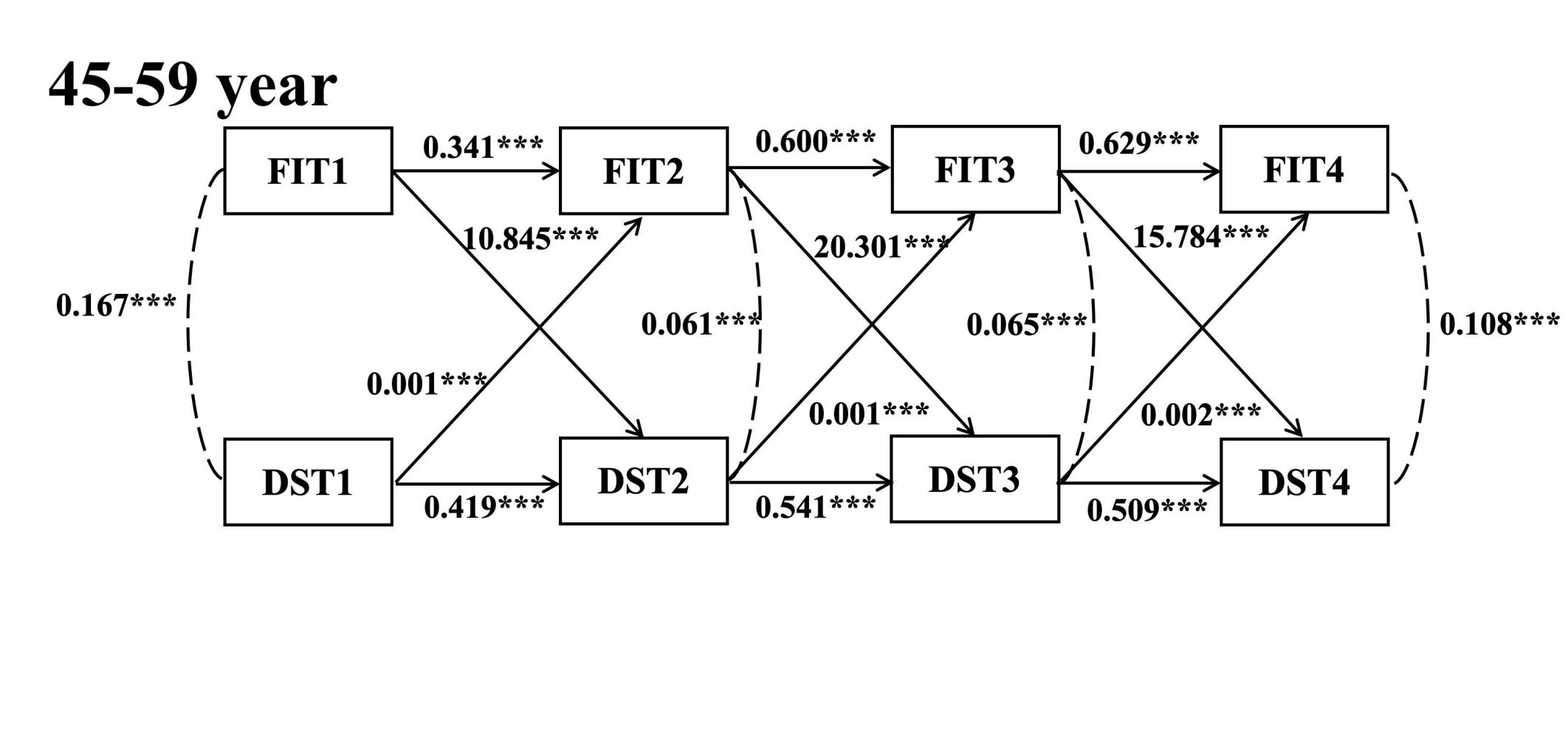


**Supplementary Figure 13.** Cross-lagged Model for Frailty and Depressive Symptoms in persons aged 45 - 59 years.

**Note:** FIT1 = frailty index in 2011; FIT2 = frailty index in 2013; FIT3 = frailty index in 2015; FIT4 = frailty index in 2018; DST1 = depressive symptoms in 2011; DST2 = depressive symptoms in 2013; DST3 = depressive symptoms in 2015; DST4 = depressive symptoms in 2018; Adjusting covariates: Education

; Marital status; Smoking; Drinking;Gender; ***:*P*<0.001;


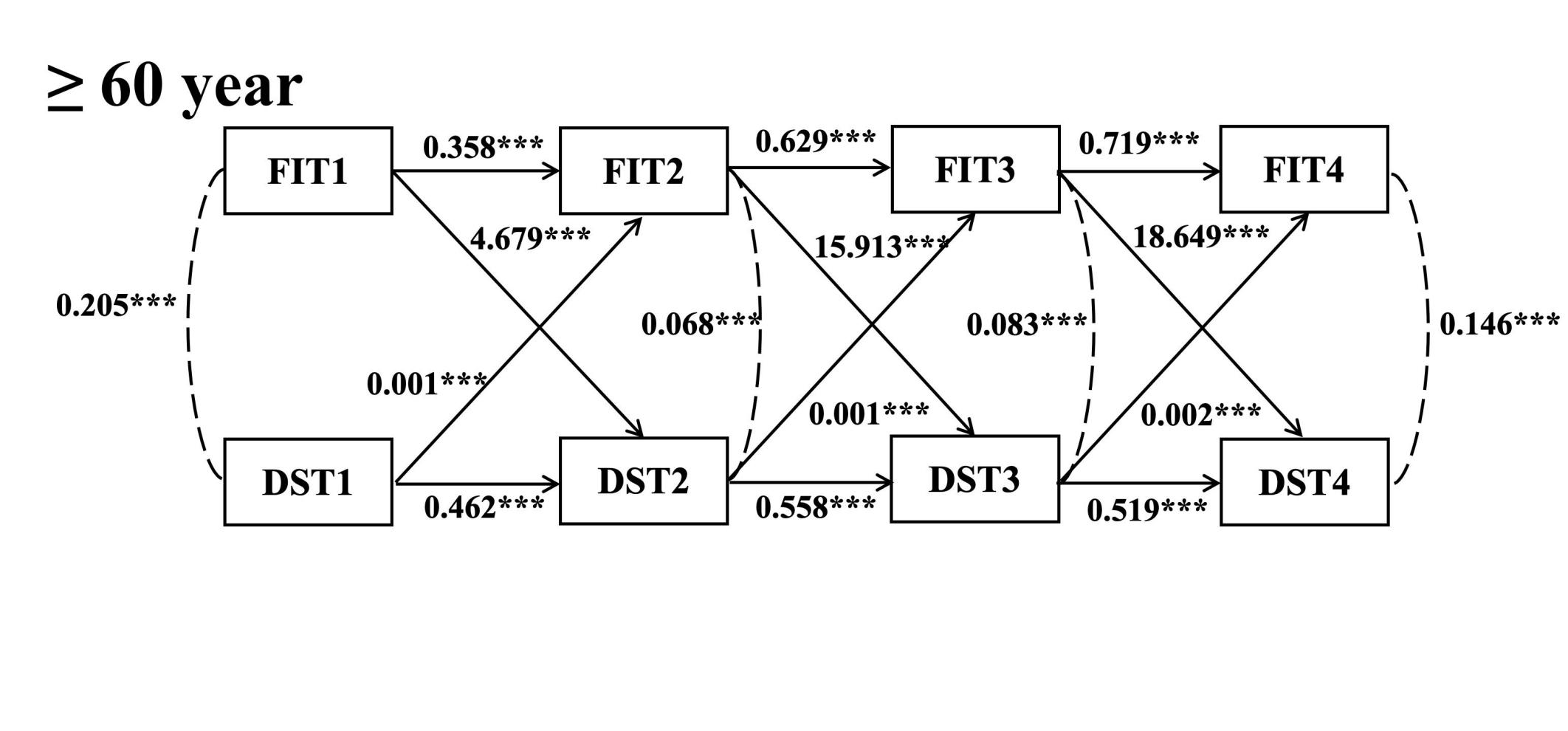


**Supplementary Figure 14.** Cross-lagged Model for Frailty and Depressive Symptoms in persons aged ≥ 60 years.

**Note:** FIT1 = frailty index in 2011; FIT2 = frailty index in 2013; FIT3 = frailty index in 2015; FIT4 = frailty index in 2018; DST1 = depressive symptoms in 2011; DST2 = depressive symptoms in 2013; DST3 = depressive symptoms in 2015; DST4 = depressive symptoms in 2018; Adjusting covariates: Education

; Marital status; Smoking; Drinking;Gender; ***:*P*<0.001;

**
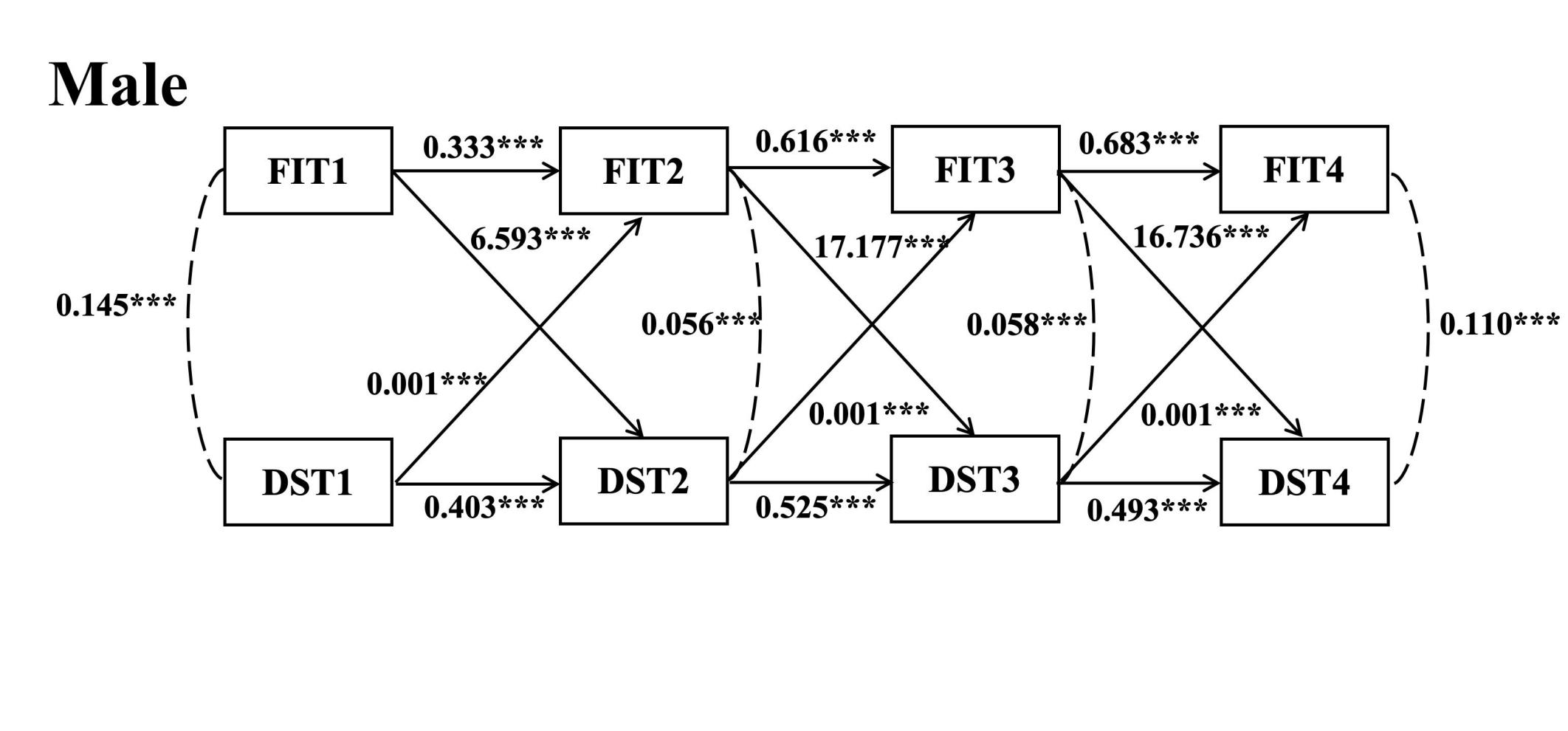
**

**Supplementary Figure 15.** Cross-lagged Model for Frailty and Depressive Symptoms in Male.

**Note:** FIT1 = frailty index in 2011; FIT2 = frailty index in 2013; FIT3 = frailty index in 2015; FIT4 = frailty index in 2018; DST1 = depressive symptoms in 2011; DST2 = depressive symptoms in 2013; DST3 = depressive symptoms in 2015; DST4 = depressive symptoms in 2018; Adjusting covariates: Age; Education; Marital status; Smoking; Drinking; ***:*P*<0.001;

**
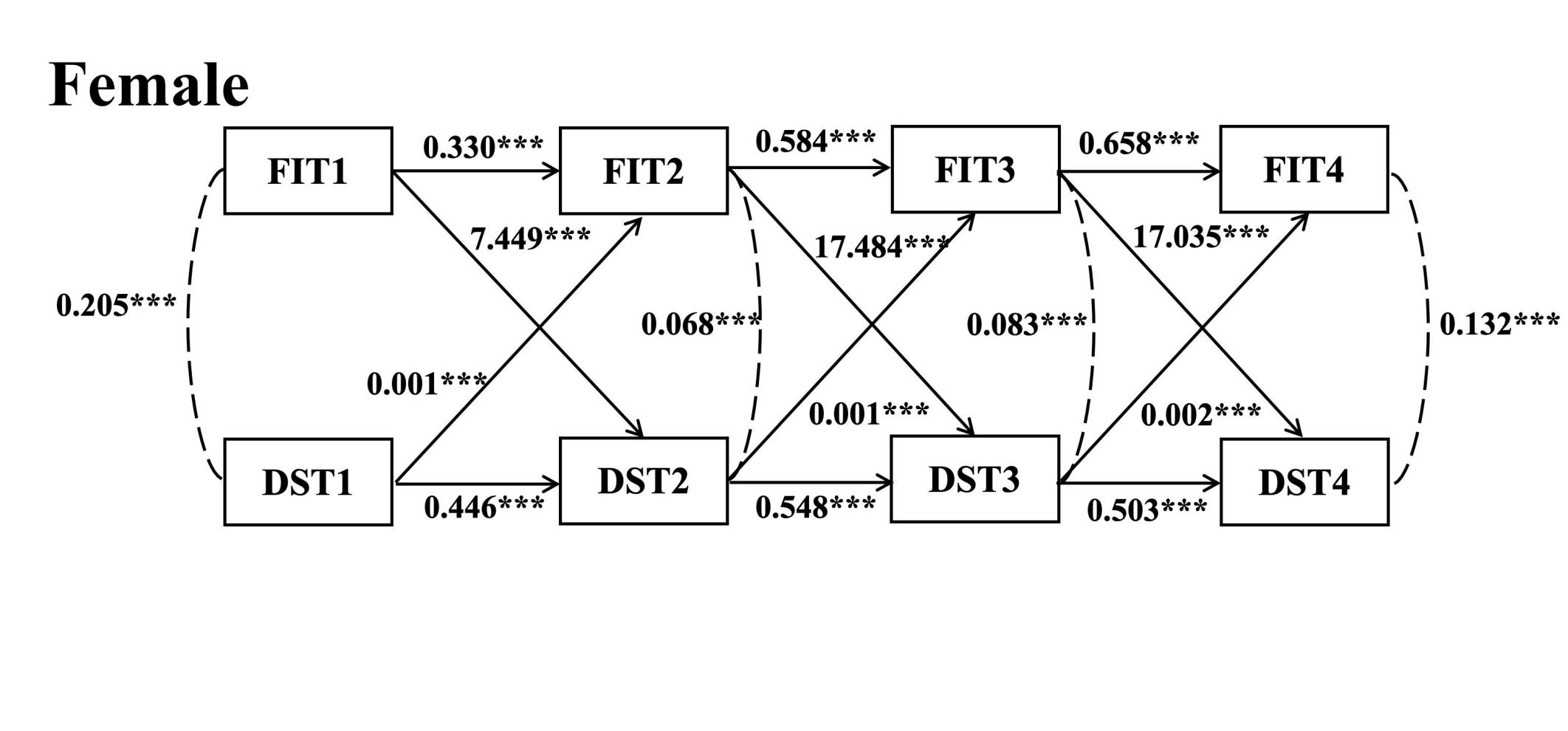
**

**Supplementary Figure 16.** Cross-lagged Model for Frailty and Depressive Symptoms in Female.

**Note:** FIT1 = frailty index in 2011; FIT2 = frailty index in 2013; FIT3 = frailty index in 2015; FIT4 = frailty index in 2018; DST1 = depressive symptoms in 2011; DST2 = depressive symptoms in 2013; DST3 = depressive symptoms in 2015; DST4 = depressive symptoms in 2018; Adjusting covariates: Age; Education; Marital status; Smoking; Drinking; ***:*P*<0.001;
